# Inhibitory Evaluation of Sulfonamide Chalcones on β-Secretase and Acylcholinesterase

**DOI:** 10.3390/molecules18010140

**Published:** 2012-12-21

**Authors:** Jae Eun Kang, Jung Keun Cho, Marcus J. Curtis-Long, Hyung Won Ryu, Jin Hyo Kim, Hye Jin Kim, Heung Joo Yuk, Dae Wook Kim, Ki Hun Park

**Affiliations:** 1 Division of Applied Life Science (BK21 Program), IALS, Gyeongsang National University, Jinju 660-701, Korea; 2 Graduate Program in Biochemistry and Biophysics, Brandeis University, 415 South Street, Waltham, MA 02453, USA; 3 Chemical Safety Division, National Academy of Agricultural Sciences, RDA, Suwon 441-701, Korea

**Keywords:** sulfonamide chalcone, Alzheimer’s disease (AD), β-secretase, acetylcholinesterase (AChE), butyrylcholinesterase (BChE), mixed inhibition

## Abstract

The action of β-secretase (BACE1) is strongly correlated with the onset of Alzheimer’s disease (AD). Aminochalcone derivatives were examined for their ability to inhibit BACE1. Parent aminochalcones showed two digit micromolar IC_50_s against BACE1. Potency was enhanced 10-fold or more by introducing benzenesulfonyl derivatives to the amino group: **1** (IC_50_ = 48.2 μM) *versus* **4a** (IC_50_ = 1.44 μM) and **2** (IC_50_ = 17.7 μM) *versus* **5a** (IC_50_ = 0.21 μM). The activity was significantly influenced by position and number of hydroxyl groups on the chalcone B-ring: 3,4-dihydroxy **5a** (IC_50_ = 0.21 μM) > 4-hydroxy **4a** (IC_50_ = 1.44 μM) > 2,4-dihydroxy **6** (IC_50_ = 3.60 μM) > 2,5-dihydroxy **7** (IC_50_ = 16.87 μM) > des hydroxy **4b** (IC_50_ = 168.7 μM). Lineweaver-Burk and Dixon plots and their secondary replots indicate that compound **5a** was a mixed inhibitor with reversible and time-dependent behavior. Potent BACE1 inhibitors **4a,c,f**, **5a**–**c** showed moderate inhibition against two other enzymes implicated in AD pathogenesis, acetylcholinesterase (AChE) and butyrylcholinesterase (BChE), with IC_50_s ranging between 56.1 ~ 95.8 μM and 19.5 ~ 79.0 μM, respectively.

## 1. Introduction

As life expectancy grows, rendering elderly people more common in society, age related diseases have become increasingly prevalent, so it is no surprise that incidences of dementia are consequentially growing yearly. Alzheimer’s disease (AD) is the major cause of dementia (1 in 8 of people over 65 has AD in the USA) [1]. This disease involves a progressive loss of neurons in the hippocampus and cortex which leads to serious loss of global cognitive ability. AD manifests itself in the brain by loss of dendrites and axons, myelin reduction, shrinkage and finally neuronal death [2]. At the biochemical level, there is a heightened presence of extra neuronal aggregation of plaques composed of β-amyloid (Aβ) peptide [3] and neurofibrillary tangles [4]. Aβ peptides are derived from a sequential proteolytic cleavage of amyloid precursor protein (APP) by the aspartate proteases, β and γ-secretase. β-Secretase (BACE1) was discovered by the four independent groups [5,6,7,8]. The Aβ hypothesis for AD started from that endogenous BACE1 activity is increased in sporadic AD brain [9,10,11]. BACE1 is uniquely able to process APP and thus form Aβ-peptides, because BACE1 knock-out mice are unable to form Aβ peptides [12]. It has now been clearly established that overproduction of Aβ by BACE1 results in toxic fibrils causing neurodegeneration.

Intriguingly, reduced AChE expression is another common feature of AD patients. However, higher levels of AChE are observed around β-amyloid plaques [13]. Thus AChE is another good target for AD treatment and it is generally accepted that AChE is associated with β-amyloid plaques. AChE catalyzes the hydrolysis of the neurotransmitter acetylcholine into choline, silencing the signal which is carried by acetylcholine [14]. There is redundancy in this system because a related enzyme, BChE can replace AChE and hydrolyze acetylcholine. Because of their similar hydrolytic activities, AChE/BChE overexpression around plaques can lead to reduced neurotransmitter levels, and thus it is thought that AChE/BChE inhibition may alleviate some AD symptoms by prolonging the half lives of neurotransmitters.

**Figure 1:**
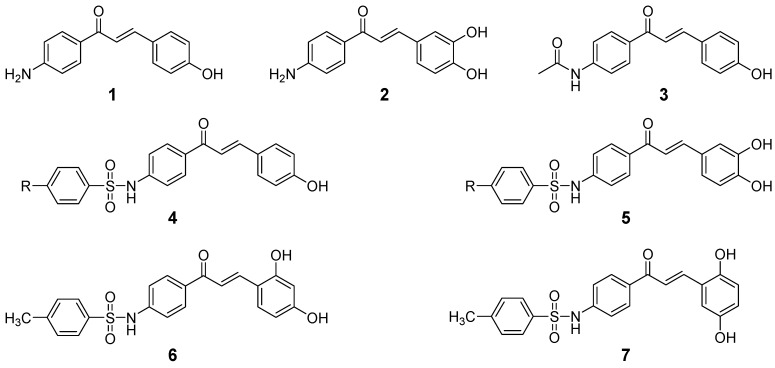
Chemical structures of chalcone derivatives.

Previous work in designing inhibitors for BACE1 has centered on peptide-derived structures, which act as transition state analogs based on the amino acid sequences at the cleavage site of APP by BACE1 [15]. These species showed nanomolar IC_50_s against BACE1 or better, but their viability as drug candidates is minimal because of their high hydrophilicity. This is because the inhibitor must possess sufficient lipophilicity to traverse two lipid bilayers to reach BACE1, as it is localized in the trans Golgi network/endosomal lumen [16]. We have previously reported the synthesis of a series of sulfonamide chalcones and their biological applications. These species showed anti-tumorigenic effects [17] as well as inhibitory activity against α-glucosidase [18]. Sulfonamide chalcones also acted as ion channel blockers [19]. In this manuscript, we disclose that novel sulfonamide chalcone derivatives can inhibit BACE1 at nanomolar levels. Their inhibitory mechanisms were assessed using Lineweaver-Burk and Dixon plots. Importantly, the most potent BACE1 inhibitors were also shown to have inhibitory activity against AChE and BChE, other key players in AD progression. Our aim was to find the structural features of chalcone with inhibitory activity for target enzymes (BACE1 and AChE). We have studied the influence on target enzymes inhibitions of each of these elements; *N*-free aminochalcone (**1**–**2**, Figure 1), *N*-acetyl aminochalcone (**3**, Figure 1), and *N*-sulfonamide chalcones (**4**–**7**, Figure 1) that have different position and number of hydroxyl groups on the chalcone B-ring.

## 2. Results and Discussion

### 2.1. Chemistry

**Scheme 1:**
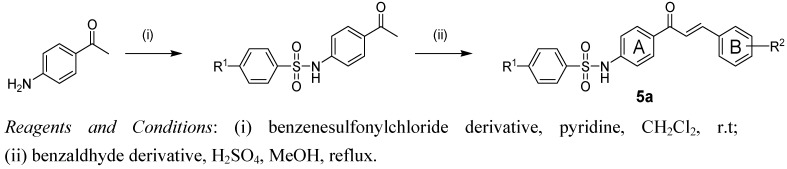
Synthesis of chalcone derivatives.

The typical synthetic procedure for sulfonamide chalcones **4**–**7** is described in Scheme 1 [11]. *N*-Sulfonylaminoacetophenone was easily prepared by treatment of aminoacetophenone with the appropriate benzenesulfonyl chloride in the presence of pyridine. The sulfonamide chalcones **1**–**7** in Table 1 were obtained by Claisen-Schmit condensation of the appropriate benzaldehyde and sulfonated aminoacetophenone in presence of a catalytic amount of H_2_SO_4_.

**Table 1:** Inhibitory effects of compounds on BACE1 activities.

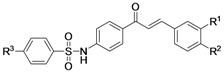			
Compound	R^1^ R^2^ R^3^	IC_50_ (mM) ^a^	*K*_i_ (μM) ^b^
**1**		48.2 ± 1.2	43.8
**2**		17.7 ± 0.8	10.5
**3**		>200	NT
**4a**	H OH CH_3_	1.44 ± 0.2	0.56
**4b**	H H CH_3_	168.7 ± 2.4	NT
**4c**	H OH H	6.28 ± 0.7	5.83
**4d**	H OH OH	2.88 ± 0.5	1.49
**4e**	H OH OCH_3_	79.3 ± 2.3	75.2
**4f**	H OH NH_2_	5.58 ± 1.4	4.19
**4g**	H OH NO_2_	107.4 ± 2.7	94.8
**4h**	H OH F	119.6 ± 3.2	NT
**5a**	OH OH CH_3_	0.21 ± 0.02	0.07
**5b**	OH OH H	4.59 ± 1.5	3.78
**5c**	OH OH OH	0.62 ± 0.03	0.56
**5d**	OH OH NH_2_	0.69 ± 0.04	0.69
**5e**	OH OH NO_2_	101.3 ± 2.4	92.4
**5f**	OH OH F	8.95 ± 1.0	10.8
**6**		3.60 ± 0.3	1.89
**7**		16.87 ± 0.8	11.7

^a^ All compounds were examined in a set of experiments repeated three times; Error is standard deviation. IC_50_ values of compounds represent the concentration that caused 50% enzyme activity loss; ^b^ Values of inhibition constant.

### 2.2. Biological Activities

#### β-Secretase Inhibition Activities

The ability of compounds **1**–**7** to inhibit the cleavage of APP to Aβ was assessed using a fluorescent resonance energy transfer (FRET) peptide cleavage assay [9]. In this assay, BACE1-catalyzed proteolysis of a synthetic oligopeptide substituted with a fluorescent dye and a quencher leads to an increase in fluorescence by breaking the tether between the dye and the quencher. The FRET oligopeptide substrate, Rhodamine-EVNLDAEFK-Quencher, and recombinant human BACE1 were purchased from RD System, Inc. (WI, USA) Morin was used as a positive control (IC_50_ = 12.3 μM). The assay was carried out according to the supplied manual with small modifications. The concentration of compound required to inhibit the enzyme 50% (IC_50_) values (μM) are presented in Table 1. These data showed that most sulfonamide containing compounds are good inhibitors of BACE1. Sulfonamide chalcones showed at least 10-fold greater potency than the des sulfonamido analogues (compounds **1** and **2**). For example, sulfonamide chalcone **5a** (IC_50_ = 0.21 μM) was 70-fold effective than the analogue bearing an unsubstituted amine, **2** (IC_50_ = 17.7 μM). A similar trend was observed for sulfonamide chalcone **4a** (IC_50_ = 1.44 μM) and amino chalcone **1** (IC_50_ = 48.2 μM). The inhibition activity was significantly influenced by position and number of hydroxyl groups on the chalcone B-ring: 3,4-dihydroxy **5a** (IC_50_ = 0.21 μM) > 4-hydroxy **4a** (IC_50_ = 1.44 μM) > 2,4-dihydroxy **6** (IC_50_ = 3.60 μM) > 2,5-dihydroxy **7** (IC_50_ = 16.87 μM) > deshydroxy **4b** (IC_50_ = 168.7 μM). Acylation and alkylation at nitrogen resulted in considerable loss of potency (supplementary material). For example, acetoamide chalcone **3** was inactive at concentrations as high as 200 μM.

**Figure 2:**
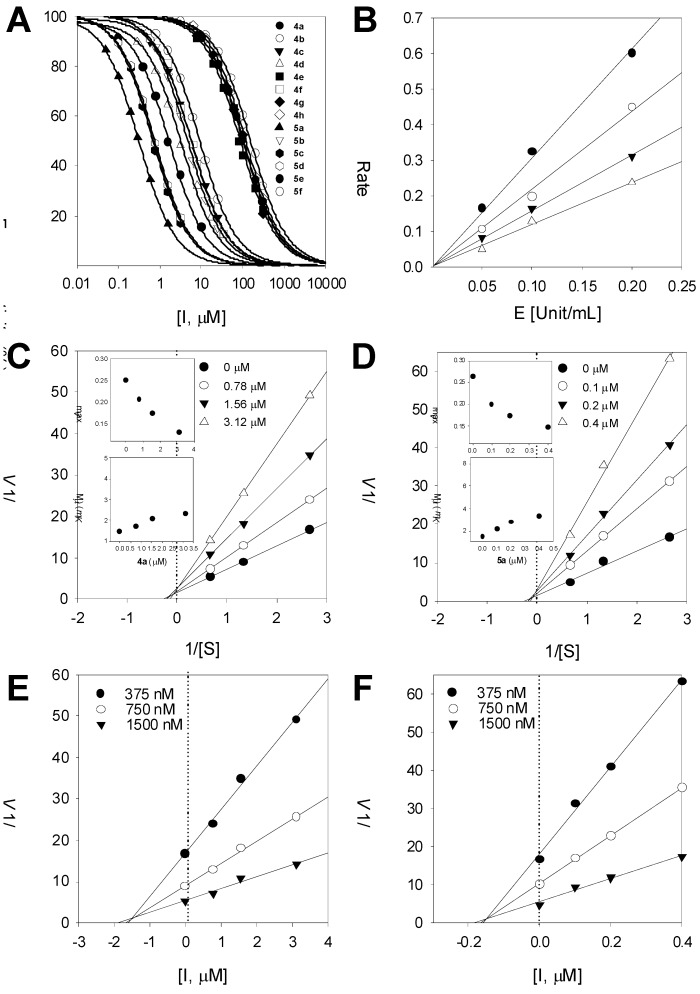
(**A**) Effect of compounds **4a**–**5f** on the hydrolytic activity of BACE1. (**B**) The hydrolytic activity of BACE1 as function of enzyme concentrations at different concentrations of compound **5a**. (**C** and **F**) Mechanistic analysis of the inhibition of BACE1 by sulfonamide chalcones **4a** and **5a**. (**C** and **D**) Lineweaver-Burk plots for the inhibition of compounds **4a** and **5a** on the hydrolytic activity of BACE1. (Inset) *K*_m_ values as a function of the concentrations of **4a** and **5a**. Dependence of the values of *V*_max_ on the concentration of **4a** and **5a**. (**E** and **F**) Dixon plots for the inhibition of compounds **4a** and **5a** on BACE1 catalyzed proteolysis of substrate.

The potency of these inhibitors was affected by subtle changes in structure. A general better inhibition was observed when the benzenesulfonyl ring bears electron donating group (CH_3_, OH, NH_2_, OCH_3_) in para position, but the inhibition was affected by electron donating potential as well as size of functionality: **4d** (OH, IC_50_ = 2.88 μM) *versus* **4e** (R^3^=OCH_3_, IC_50_ = 79.3 μM). Consistent with this trend, the strongly electron withdrawing groups, *p*-fluoro and *p*-nitro, were the least potent inhibitors (IC_50_ = 119.6 and 107.4 μM respectively; Figure 2A). The **5**-series, bearing a 3,4-dihydroxy group in the B ring of the chalcone, was in general more potent than the corresponding 4-hydroxy derivatives. Interestingly, the relationship between activity and substituent on the sulfonamide function held steady. Smaller electron donating groups (CH_3_, OH, NH_2_) were more favored than larger species such as OCH_3_ and electron withdrawing functions such as NO_2_.

### 2.3. Inhibitory Kinetics

We examined each sulfonamide compound **4a**–**5f** for its inhibition of BACE1 (Figure 2). As the concentrations of the inhibitors were augmented, the residual enzyme activity drastically diminished (Figure 2A). The inhibition of BACE1 by compounds **4a** (*K*_i_ = 0.56 μM) and **5a** (*K*_i_ = 0.07 μM), the most potent inhibitors, are illustrated in Figure 2, representatively. Raising the concentrations of the inhibitors drastically lowered residual enzyme activity. Plots of residual enzyme activity *versus* enzyme concentration at different concentrations of compound **5a** gave a family of straight lines with a *y*-axis intercept of 0; this indicates that **5a** is a reversible inhibitor (Figure 2B). We then further characterized the inhibitory mechanism of the synthesized chalcone derivatives. This was done using both Lineweaver-Burk and Dixon plots. These double-reciprocal plots yield a family of lines with different slopes and intercepts. They intersected to the left of the vertical axis and above the horizontal axis, indicating that **5a** is a mixed inhibitor (Figure 2D). The mixed behavior of **5a** was also proved by showing increasing concentrations of inhibitor resulted in increased *K*_m_ values and diminished *V*_max_ The *K*_i_ of **5a** was determined to be 70 nM (Table 1) using a Dixon plot (Figure 2F). Compound **4a** (*K*_i_ = 0.56 μM) also showed mixed type behavior with a similar kinetic pattern to **5a** (Figure 2E,F). The rest of tested compounds were also mixed inhibitors.

**Figure 3:**
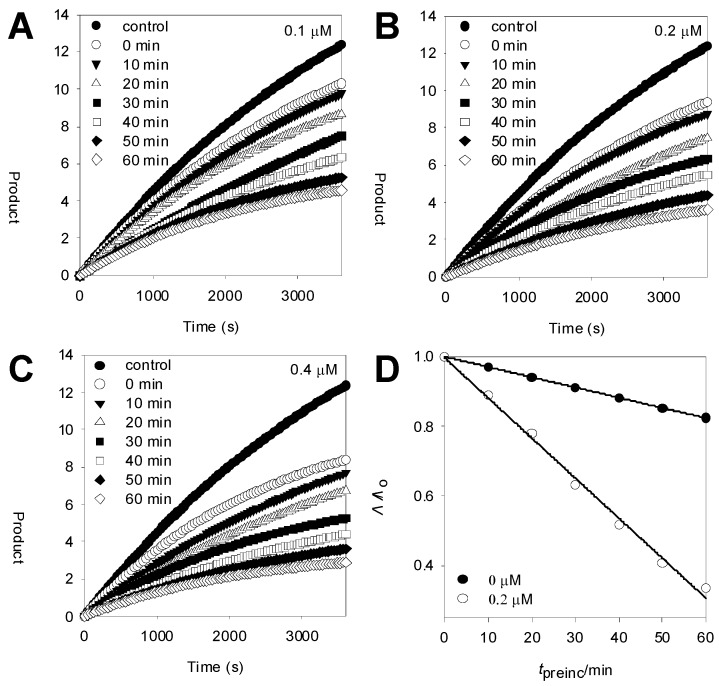
(**A**–**C**) Time-dependent inhibition of BACE1 in the presence of 0.1, 0.2, 0.4 μM compound **5a**; (**D**) Decrease in slopes of the lines of panel E as a function of time (●, 0 μM; ○, 0.2 μM).

To further investigate the inhibition mechanism, time dependence of BACE1inhibition by compound **5a** was subsequently probed. The enzyme was preincubated with substrate (between 0 and 60 min) and the residual enzyme activity was measured by calculating the initial velocity (V_i_) of the hydrolysis (Figure 3B). Progress curves were continued to be run up for 3,600 s. These data showed continued decrease in activity to the final steady state rate. Secondary replots of V_i_/V_0_ after preincubation with inhibitor compared to similar incubation times without inhibitor show that a significant time dependence in inhibition is observed. Thus **5a** is a slow binding inhibitor of this enzyme (Figure 3D) [20].

Since AChE and BChE have also been implicated in the onset of AD we were keen to see if our compounds could inhibit these enzymes as well. Clearly an inhibitor capable of alleviating numerous etiological factors simultaneously could have more benefit than a compound which was highly selective for one enzyme. Our most potent BACE1 inhibitors **4a,c,f**, **5a**–**c** were thus assessed for inhibition of AChE and BChE. As shown Table 2, many of our sulfonamide chalcones exhibited a moderate degree of cholinesterase inhibition, with IC_50_s ranging from 56.1 ~ 95.8 μM for AChE and 19.5 ~ 79.0 μM for BChE. Further analysis showed all ChE inhibitors were reversible and exhibited mixed inhibition kinetics (Table 2 and Supplementary Materials).

**Table 2:** Inhibitory effects of selected sulfonamide chalcones on the cholinesterasesAChE and BChE.

Compound	IC_50_^a^ (μM)	*K*_i_ (μM)^b^	IC_50_^a^ (μM)	*K*_i_ (μM)^b^
	erythrocytes AChE		equine serum BChE	
**4a**	56.1	40.4	47.4	25.3
**4c**	83.3	80.3	75.0	72.2
**4f**	95.8	61.5	34.3	19.0
**5a**	75.9	55.8	19.5	9.8
**5b**	75.4	57.2	79.0	63.5
**5c**	57.4	47.6	24.7	26.6
**Eserin**	0.15	NT ^c^	3.7	NT

^a^ All compounds were examined in a set of experiments repeated three times; Error is standard deviation. IC_50_ values of compounds represent the concentration that caused 50% enzyme activity loss; ^b^ Values of inhibition constant; ^c^ NT is not tested.

## 3. Experimental

### 3.1. General

Test chromatographic separations were carried out by Thin-layer chromatography (TLC) (E. Merck Co., Darmstadt, Germany), using commercially available glass plate pre-coated with silica gel and visualized under UV at 254 and 366 nm or with *p*-anisaldehyde staining reagent. Column chromatography was carried out using 230–400 mesh silica gel (kieselgel 60, Merck). Melting points were measured on a Thomas Scientific capillary melting point apparatus (Electrothermal 9300, Swedesboro, NJ, USA) and are uncorrected. Infra red (IR) spectra were recorded on a Bruker IFS66 (Bruker, Karlsruhe, Germany) infrared Fourier transform spectrophotometer (KBr) and UV spectra were measured on a Beckman DU650 spectrophotometer (Beckman Coulter, Fullerton, CA, USA). ^1^H- and ^13^C-NMR along with 2D-NMR data were obtained on a Bruker AM 500 (^1^H-NMR at 500 MHz, ^13^C-NMR at 125 MHz) spectrometer (Bruker, Karlsruhe, Germany) in CDCl_3_, acetone-*d_6_*, DMSO-*d_6_*, and CD_3_OD. EIMS was obtained on a JEOL JMS-700 mass spectrometer (JEOL, Tokyo, Japan). All the reagent grade chemicals were purchased from Sigma Chemical Co. (St. Louis, MO, USA).

### 3.2. Synthesis of Chalcone Derivatives

To a solution of aminoacetophenone derivative (50 mmol) in methanol (100 mL) was added benzaldehyde derivative (60 mmol) with a catalytic amount of H_2_SO_4_. The reaction mixture was refluxed for 24 h and neutralized with 1.5% NaOH (50 mL). The mixture was extracted with EtOAc, and dried over anhydrous Na_2_SO_4_. After evaporation of EtOAc layer, the residue was purified by silica gel column chromatography eluting with hexane/EtOAc to give the pure chalcone derivatives.

*4′-Amino-4-hydroxychalcone* (**1**). m.p. 79–80 °C; IR (KBr): 3510, 1665 cm^−1^; ^1^H-NMR (300 MHz; MeOD) δ 6.44 (2H, dd, *J_1_* = 6.9, *J_2_* = 1.9 Hz), 6.85 (2H, dd, *J_1_* = 6.9, *J_2_* = 1.8 Hz), 7.55 (3H, m), 7.68 (1H, d, *J* = 15.5 Hz), 7.91 (2H, dd, *J_1_* = 7.9, *J_2_* = 2.0 Hz); ^13^C-NMR (75 MHz; MeOD) δ 113.1, 115.5, 118.5, 126.5, 126.8, 130.1, 131.0, 143.4, 153.8, 159.7 and 189.1; EIMS *m*/*z* 239 [M^+^]; HREIMS *m/z* 239.0948 [M^+^] (calculated for C_15_H_13_NO_2_, 239.0946).

*4′-Amino-3,4-dihydroxychalcone* (**2**). m.p. 184–185 °C; IR (KBr): 3400, 1780 cm^−1^; ^1^H-NMR (300 MHz; MeOD) δ 6.57 (2H, dd, *J_1_* = 8.8, *J_2_* = 1.9 Hz), 6.83 (2H, d, *J* = 9.0 Hz), 7.08 (1H, dd, *J_1_* = 8.2, *J_2_* = 2.0 Hz), 7.18 (1H, d, *J* = 2.0 Hz), 7.50 (1H, d, *J* = 15.4 Hz), 7.62 (1H, d, *J* = 15.5 Hz), 7.90 (2H, m); ^13^C-NMR (75 MHz; MeOD) δ 113.8, 114.2, 115.2, 118.5, 121.8, 126.5, 127.4, 130.9, 143.7, 145.4, 148.1, 153.9 and 189.1; EIMS *m*/*z 255* [M^+^]; HREIMS *m/z* 255.0896 [M^+^] (calculated for C_15_H_13_NO_3_, 255.0895).

*4′-Acetamido-4-hydroxychalcone* (**3**). m.p. 202 °C EIMS *m/z* 281 [M]^+^; HREIMS *m/z* 281.1052, (calcd for C_17_H_15_NO_3_, 281.1052); ^1^H-NMR (300 MHz, acetone-*d_6_*) *δ* 2.05 (3H, s), 6.93 (2H, d, *J* = 8.7 Hz), 7.72 (4H, m), 7.81 (2H, d, *J* = 8.5 Hz), and 8.11 (2H, d, *J* = 8.7 Hz); ^13^C-NMR (75 MHz) *δ* 23.5, 115.8, 118.3, 118.7, 126.8, 129.5, 130.5, 133.3, 143.5, 159.9, 168.5, 175.7, and 187.4.; EIMS *m*/*z 281* [M^+^]; HREIMS *m/z* 281.1053 [M^+^] (calculated for C_15_H_13_NO_3_, 281.1052).

*4'-(4-Toluenesulfonamide)-4-hydroxychalcone* (**4a**). m.p.: 105–107 °C; IR (KBr): 3520, 1675 cm^−1^; ^1^H-NMR (300 MHz; MeOD) δ 2.25 (3H, s), 6.82 (2H, d, *J* = 8.6 Hz), 7.23 (4H, m), 7.42 (1H, d, *J* = 15.5 Hz), 7.51 (2H, d, *J* = 8.6 Hz), 7.66 (1H, d, *J* = 15.5 Hz), 7.73 (2H, d, *J* = 8.3 Hz), 7.90 (2H, dd, *J_1_* = 8.7, *J_2_* = 2.0 Hz); ^13^C-NMR (75 MHz; MeOD) δ 20.1, 115.6, 118.0, 118.5, 126.4, 126.9, 129.4, 129.8, 130.5, 133.5, 136.6, 142.4, 144.1, 145.2, 160.2 and 189.7 (Found; C, 67.16; H, 4.87; N, 3.56; C_22_H_19_NO_4_S requires C, 67.14; H, 4.88; N, 3.57%).

*4′-(4-Toluenesulfonamido)-chalcone* (**4b**). m.p.: 160–161 °C; ^1^H-NMR (300 MHz; CDCl_3_) *δ* 2.38 (3H, s), 7.25 (2H, d, *J* = 1.92 Hz), 7.28 (2H, s), 7.38 (1H, m), 7.42 (2H, m), 7.50 (1H, d, *J* = 15.7 Hz), 7.64 (1H, m), 7.78 (3H, m), 7.88 (1H, s), 7.94 (2H, d, *J* = 8.70 Hz); ^13^C-NMR (75 MHz; CDCl_3_) *δ* 21.6, 119.0, 121.5, 127.3, 128.5, 129.9, 130.2, 130.7, 134.0, 134.8, 135.8, 141.2, 144.5, 144.9, 189.1; EIMS *m*/*z* 377 [M^+^]; HREIMS *m/z* 377.1083 [M^+^] (calculated for C_22_H_19_NO_3_S, 377.1086).

*4′-(Benzenesulfonamide)-4-hydroxychalcone* (**4c**). m.p. 206−208 °C; IR (KBr): 3535, 1655 cm^−1^; ^1^H-NMR (300 MHz; MeOD) δ 6.84 (2H, d, *J* = 8.6 Hz), 7.27 (2H, d, *J* = 8.7 Hz), 7.55 (6H, m), 7.71 (1H, d, *J* = 15.5 Hz), 7.88 (2H, d, *J* = 8.6 Hz), 7.96 (2H, d, *J* = 8.7 Hz); ^13^C-NMR (75 MHz; MeOD) δ 115.5, 117.9, 118.5, 126.3, 126.8, 128.4, 129.7, 130.5, 132.8, 133.6, 139.7, 142.3, 145.2, 160.3 and 189.6; EIMS *m*/*z* 379 [M^+^]; HREIMS *m/z* 379.0873 [M^+^] (calculated for C_21_H_17_NO_4_S, 379.0878).

*4′-(4-Hydroxybenzenesulfonamide)-4-hydroxychalcone* (**4d**). m.p. 102−103 °C; IR (KBr): 3525, 1670 cm^−1^; ^1^H-NMR (300 MHz; Acetone-*d_6_*) δ 6.94 (3H, m), 7.37 (1H, d, *J* = 8.7 Hz), 7.67 (3H, m), 7.77 (2H, d, *J* = 8.8 Hz), 8.04 (2H, m), 8.70 (3H, s); ^13^C-NMR (75 MHz; Acetone-*d_6_*) δ 115.6, 115.8, 118.4, 118.5, 126.8, 129.6, 128.9, 130.3, 130.7, 133.7, 142.4, 144.0, 159.9, 161.5 and 187.6; EIMS *m*/*z* 395 [M^+^]; HREIMS *m/z* 395.0830 [M^+^] (calculated for C_21_H_17_NO_5_S, 395.0827).

*4′-(4-Methoxybenzenesulfonamido)-4-hydroxychalcone* (**4e**). m.p. 90−91 °C; IR (KBr): 3400, 1780 cm^−1^; ^1^H-NMR (300 MHz; MeOD) δ 3.84 (3H, s), 6.93 (2H, d, *J* = 1.9 Hz), 7.06 (2H, d, *J* = 2.1 Hz), 7.39 (2H, d, *J* = 1.9 Hz), 7.85 (4H, m), 8.05 (2H, d, *J* = 1.8 Hz), 8.07 (2H, d, *J* = 1.9 Hz); ^13^C-NMR (75 MHz; MeOD) δ 55.2, 114.3, 115.9, 18.5, 126.8, 129.3, 129.9, 130.7, 131.3, 133.8, 142.3, 143.9, 159.9, 163.3 and 187.5; EIMS *m*/*z 409* [M^+^]; HREIMS *m/z* 409.0985 [M^+^] (calculated for C_22_H_19_NO_5_S, 409.0984).

*4′-(4-Aminobenzenesulfonamide)-4-hydroxychalcone* (**4f**). m.p. 216−217 °C; IR (KBr): 3526, 1663 cm^−1^; ^1^H-NMR (300 MHz; Acetone-*d_6_*) δ 6.70 (2H, d, *J* = 8.7), 6.93 (2H, d, *J* = 8.6 Hz), 7.37 (2H, d, *J* = 8.7 Hz), 7.61 (2H, d, *J* = 8.8 Hz), 7.70 (4H, m) and 8.06 (2H, d, *J* = 8.7 Hz); ^13^C-NMR (75 MHz; Acetone-*d_6_*) δ 113.1, 115.9, 118.2, 118.6, 125.7, 126.9, 129.2, 129.8, 130.6, 133.4, 142.9, 143.9, 153.1, 159.9 and 187.6; EIMS *m*/*z* 394 [M^+^]; HREIMS *m/z* 394.0985 [M^+^] (calculated for C_21_H_18_N_2_O_4_S, 394.0987).

*4′-(4-Nitrobenzenesulfonamide)-4-hydroxychalcone* (**4g**). m.p. 136−138 °C; IR (KBr): 3513, 1656 cm^−1^; ^1^H-NMR (300 MHz; Acetone-*d_6_*) δ 6.92 (2H, d, *J* = 8.6 Hz), 7.42 (2H, d, *J* = 8.7 Hz), 7.68 (4H, m), 8.08 (2H, d, *J* = 8.7 Hz), 8.17 (2H, d, *J* = 8.9 Hz) and 8.41 (2H, d, *J* = 8.9 Hz); ^13^C-NMR (75 MHz; Acetone-*d_6_*) δ 116.3, 118.7, 119.3, 125.3, 126.3, 128.8, 130.5, 131.4, 134.2, 141.7, 144.6, 145.1, 150.5, 160.6 and 187.9; EIMS *m*/*z* 424 [M^+^]; HREIMS *m/z* 424.0728 [M^+^] (calculated for C_21_H_16_N_2_O_6_S, 424.0729).

*4′-(4-Fluorobenzenesulfonamide)-4-hydroxychalcone* (**4h**). m.p. 179−180 °C; IR (KBr): 3544, 1671 cm^−1^; ^1^H-NMR (300 MHz; MeOD) δ 6.84 (2H, d, *J* = 8.6 Hz), 7.26 (4H, m), 7.55 (3H, m), 7.72 (1H, d, *J* = 15.5 Hz) and 7.94 (4H, m); ^13^C-NMR (75 MHz; MeOD) δ 115.5, 115.8, 116.1, 117.9, 118.7, 126.3, 129.7, 129.9, 130.5, 133.8, 135.8, 142.1, 145.2, 160.3 and 189.6; EIMS *m/z* 397 [M^+^]; HREIMS *m/z* 397.0782 [M^+^] (calculated for C_21_H_16_FNO_4_S, 397.0784).

*4'-(4-Toluenesulfonamide)-3,4-dihydroxychalcone* (**5a**). m.p.: 179–180 °C; IR (KBr): 3480, 1685 cm^−1^; ^1^H-NMR (300 MHz; MeOD+DMSO-d_6_) δ 2.29 (3H, s), 6.78 (1H, d, *J* = 8.2 Hz), 6.94 (1H, dd, *J_1_* = 8.2, *J_2_* = 2.0 Hz), 7.10 (1H, d, *J* = 2.0 Hz), 7.19 (5H, m), 7.50 (1H, d, *J* = 15.2 Hz), 7.66 (2H, d, *J* = 8.2 Hz), 7.76 (2H, d, *J* = 8.6 Hz); ^13^C-NMR (75 MHz; MeOD+DMSO-d_6_) δ 21.4, 115.0, 115.9, 118.6, 122.0, 127.0, 129.6, 129.7, 133.6, 136.9, 142.2, 143.6, 145.0, 145.4, 148.3 and 188.7; EIMS *m*/*z* 409 [M^+^]; HREIMS *m/z* 409.0985 [M^+^] (calculated for C_22_H_19_NO_5_S, 409.0984).

*4′-(Benzenesulfonamide)-3,4-dihydroxychalcone* (**5b**). m.p. 206–208 °C; IR (KBr): 3535, 1655 cm^−1^; ^1^H-NMR (300 MHz; MeOD) δ 3.36 (3H, s), 6.82 (1H, d, *J* = 8.2 Hz), 7.02 (1H, d, *J* = 8.7 Hz), 7.09 (1H, d, *J* = 1.9 Hz), 7.27 (2H, d, *J* = 8.6 Hz), 7.46 (1H, d, *J* = 15.5 Hz), 7.56 (2H, m), 7.64 (1H, d, *J* = 15.5 Hz), 7.87 (1H, d, *J* = 8.7 Hz), 7.95 (1H, d, *J* = 8.6 Hz); ^13^C-NMR (75 MHz; MeOD) δ 113.5, 116.8, 117.2, 120.4, 121.3, 127.3, 127.9, 129.1, 129.2, 130.7, 132.0, 139.7, 143.5, 145.2, 146.5, 147.2 and 189.7; EIMS *m*/*z 395* [M^+^]; HREIMS *m/z* 395.0828 [M^+^] (calculated for C_21_H_17_NO_5_S, 395.0827).

*4′-(4-Hydroxybenzenesulfonamide)-3,4-hydroxychalcone* (**5c**). m.p. 102–103 °C; IR (KBr): 3525, 1670 cm^−1^; ^1^H-NMR (300 MHz; Acetone-*d_6_*) δ 6.65 (1H, m), 6.88 (2H, m), 7.64 (3H, m), 8.04 (2H, d, *J* = 8.3 Hz); ^13^C NMR (75 MHz; Acetone-*d_6_*) δ 113.5, 115.8, 116.2, 116.8, 117.0, 117.2, 120.4, 121.3, 127.9, 128.7, 128.9, 130.7, 130.8, 132.3, 143.5, 145.2, 146.5, 147.2, 161.7, and 189.7; EIMS *m*/*z* 411 [M^+^]; HREIMS *m/z* 411.0778 [M^+^] (calculated for C_21_H_17_NO_6_S, 411.0777).

*4′-(4-Aminobenzenesulfonamide)-3,4-dihydroxychalcone* (**5d**). m.p. 216–217 °C; IR (KBr): 3526, 1663 cm^−1^; ^1^H-NMR (300 MHz; MeOD) δ 6.70 (2H, m), 6.91 (1H, m), 7.19 (1H, m), 7.36 (3H, m), 7.61 (4H, m), 8.05 (2H, m); ^13^C-NMR (75 MHz; MeOD) δ 113.1, 114.9, 115.5, 118.2, 118.7, 122.3, 125.7, 127.5, 129.2, 129.8, 133.4, 142.8, 144.2, 145.4, 148.0, 153.0 and 187.6; EIMS *m*/*z 410* [M^+^]; HREIMS *m/z* 410.0937 [M^+^] (calculated for C_21_H_16_N_2_O_5_S, 410.0936).

*4′-(4-Nitrobenzenesulfonamide)-3,4-dihydroxychalcone* (**5e**). m.p. 136–138 °C; IR (KBr): 3513, 1656 cm^−1^; ^1^H-NMR (300 MHz; MeOD) δ 6.90 (1H, d, *J* = 8.2 Hz), 7.19 (1H, dd, *J_1_* = 2.0, *J_2_* = 1.9 Hz), 7.31 (1H, d, *J* = 1.7 Hz), 7.42 (2H, d, *J* = 8.7 Hz), 7.61 (2H, m), 8.07 (2H, d, *J* = 8.7 Hz), 8.17 (2H, d, *J* = 8.9 Hz), 8.41 (2H, d, *J* = 8.9 Hz); ^13^C-NMR (75 MHz; MeOD) δ 114.9, 115.5, 118.6, 119.5, 122.3, 124.5, 127.4, 128.6, 129.9, 134.8, 141.1, 144.5, 145.1, 145.5, 148.1, 150.5 and 187.5; EIMS *m*/*z 440* [M^+^]; HREIMS *m/z* 440.0679 [M^+^] (calculated for C_21_H_16_N_2_O_7_S, 440.0678).

*4′-(4-Fluorobenzenesulfonamide)-3,4-dihydroxychalcone* (**5f**). m.p. 136–138 °C; IR (KBr): 3513, 1656 cm^−1^; ^1^H-NMR (300 MHz; MeOD) δ 6.82 (1H, d, *J* = 8.2 Hz), 7.10 (1H, dd, *J_1_* = 1.8, *J_2_* = 1.7 Hz), 7.17 (1H, s), 7.27 (4H, m), 7.46 (1H, d, *J* = 15.5 Hz), 7.66 (1H, d, *J* = 15.4 Hz), 7.95 (4H, m); ^13^C-NMR (75 MHz; MeOD) δ 113.5, 115.8, 116.8, 117.2, 120.4, 121.3, 127.9, 128.9, 130.2, 130.7, 135.3, 143.5, 145.2, 146.5, 147.2, 166.1 and 189.7; EIMS *m*/*z 413* [M^+^]; HREIMS *m/z* 413.0734 [M^+^] (calculated for C_21_H_16_FNO_5_S, 413.0733).

*4'-(4-Toluenesulfonamide)-2,4-dihydroxychalcone* (**6**). m.p.: 105–107 °C; IR (KBr): 3520, 1675 cm^−1^; ^1^H-NMR (300 MHz; MeOD) δ 2.36 (3H, s), 6.35 (1H, m), 6.39 (2H, m), 7.33 (5H, m), 7.78 (3H, m), 7.91 (2H, m); ^13^C-NMR (75 MHz; MeOD) δ 24.3, 103.5, 108.4, 109.1, 116.8, 121.3, 127.2, 127.9,129.2, 129.3, 130.7, 136.7, 141.6, 143.5, 145.3, 159.1, 159.7 and 189.7; EIMS *m*/*z 409* [M^+^]; HREIMS *m/z* 409.0985 [M^+^] (calculated for C_22_H_19_NO_5_S, 409.0984).

*4'-(4-Toluenesulfonamide)-2,5-dihydroxychalcone* (**7**). m.p.: 105–107 °C; IR (KBr): 3520, 1675 cm^−1^; ^1^H-NMR (300 MHz; MeOD) δ 2.31 (3H, s), 6.76 (2H, d, *J* = 2.7 Hz), 7.08 (1H, d, *J* = 2.2 Hz), 7.26 (4H, m), 7.64 (1H, d, *J* = 15.9 Hz), 7.73 (2H, d, *J* = 8.3 Hz), 7.91 (2H, d, *J* = 8.7 Hz), 8.05 (1H, d, *J* = 15.7 Hz); ^13^C-NMR (75 MHz; MeOD) δ 20.1, 113.7, 116.7, 118.4, 119.4, 120.7, 122.1, 126.9, 129.4, 129.8, 130.7, 133.5, 136.6, 140.7, 142.4, 144.1, 149.9, 150.8 and 190.2; EIMS *m*/*z* 409 [M^+^]; HREIMS *m/z* 409.0985 [M^+^] (calculated for C_22_H_19_NO_5_S, 409.0984).

### 3.3. Pharmacology

#### 3.3.1. BACE1 Enzyme Assay

The assay was carried out according to the supplied manual with modifications. Briefly, a mixture of 10 μL of assay buffer (50 mM sodium acetate, pH 4.5), 10 μL of BACE1 (1.0 U/mL), 10 μL of the substrate (750 nM Rh-EVNLDAEFK-Quencher in 50 mM sodium acetate, pH 4.5), and 10 μL of sample dissolved in 50 mM ammonium bicarbonate buffer (pH 7.8) was incubated for 60 min at room temperature in the dark. The mixture was irradiated at 545 nm and the emission intensity at 590 nm was recorded [21,22]. The inhibition ratio was obtained by the following equation:

Inhibition (%) = [1 − {(*S*−*S*_0_)/(*C*−*C*_0_)}] × 100

where *C* was the fluorescence of the control (enzyme, buffer, and substrate) after 60 min of incubation, *C_0_* was the fluorescence of control at zero time, *S* was the fluorescence of the tested samples (enzyme, sample solution, and substrate) after incubation, and *S_0_* was the fluorescence of the tested samples at zero time. To allow for the quenching effect of the samples, the sample solution was added to the reaction mixture *C*, and any reduction in fluorescence by the sample was then investigated. All data are the mean of three experiments.

#### 3.3.2. Slow and Time-Dependent Inhibitory Activity

Slow and time-dependent assays and progress curves were carried out using 1 U/mL unit BACE1, and 10 μL substrate in 50 mM ammonium bicarbonate pH 7.8 at 37 °C. Enzyme activities were measured continuously for 3,600 s spectrophotometrically. To determine the kinetic parameters associated with time dependent inhibition of BACE1, progress curves for 3,600 s were obtained at one inhibitor concentration using fixed substrate concentrations [23,24]. The data were analyzed using the nonlinear regression program [Sigma Plot (SPCC Inc., Chicago, IL, USA)].

#### 3.3.3. Cholinesterase Inhibitory Activity

Cholinesterase was assayed according to standard procedures by following the hydrolysis of acetylthiocholine iodide (AtCh) or butyrylthiocholine iodide (BtCh) by monitoring formation of 5-thio-2-nitrobenzoate from 5-5'-dithiobis(2-nitrobenzoic acid) spectrometrically at 412 nm [25,26,27]. The reaction mixture contained 100 mM sodium phosphate buffer (pH 8.0), 20 μL of test sample solution and either 2 μL of human erythrocyte AChE (0.25 U/mL) or 2 μL of equine serum BChE (0.05 U/mL) solution, which were mixed and incubated for 10 min at room temperature. Assays were then initiated with the addition of 30 μL 5-5'-dithiobis(2-nitrobenzoic acid) (3.3 mM) and, respectively, 20 μL of AtCh (1 mM), and 7 μL of BtCh (1 mM). All assays were performed in triplicate. Compounds showing the highest inhibitory activities were further characterized by determining the concentration required to inhibit 50% of the enzyme activity under the assay conditions (defined as the IC_50_ value). Kinetic parameters were determined using the Lineweaver-Burk double-reciprocal-plot method at increasing concentration of substrates and inhibitors. The inhibitor eserine (Sigma-Aldrich, St. Louis, MO, USA) was used in the assays for comparison.

#### 3.3.4. Statistical Analysis

All the measurements were made in triplicate. The results were subject to variance analysis using Sigma plot. Differences were considered significant at *p* < 0.05.

## 4. Conclusions

We set out to evaluate sulfonamide chalcones as inhibitors of an enzyme which is key in the progression of AD, BACE1. Our work shows that these small molecules are actually very potent inhibitors of this enzyme. Some of these compounds possess IC_50_s in the nanomolar range. Thus our sulfonamide chalcones represent valuable lead compounds and could be useful in further experimentation. Our own SAR studies have shown that electron donating groups are favored on the sulfonamide function, whereas a 3,4-dihydroxy motif is preferred in the chalcone B ring. This information can also serve for further optimization in the future.

## Supplementary Materials

Supplementary materials can be accessed at: http://www.mdpi.com/1420-3049/18/1/140/s1.
