# Supplementary material for: Inhibitory Evaluation of Sulfonamide Chalcones on β-Secretase and Acylcholinesterase
Source: Molecules. 2012 Dec 21;18(1):140–53. doi: 10.3390/molecules18010140 (PMC6270012; doi:10.3390/molecules18010140)

Supplementary Materials

Experimental Section & Characterization Data

►Spectroscopic data of the compounds (**1–7**) -------------------------------**S2–S18**

►BACE1 kinetic data of the compounds (**1–5**) -----------------------------**S19–S22**

►Cholinesterase kinetic data of the compounds (**4a**,**f**, **5b,d**) -----**S23-S24**

Spectra 1. ^{1}H-NMR of 4'-amino-4-hydroxychalcone (**1**).

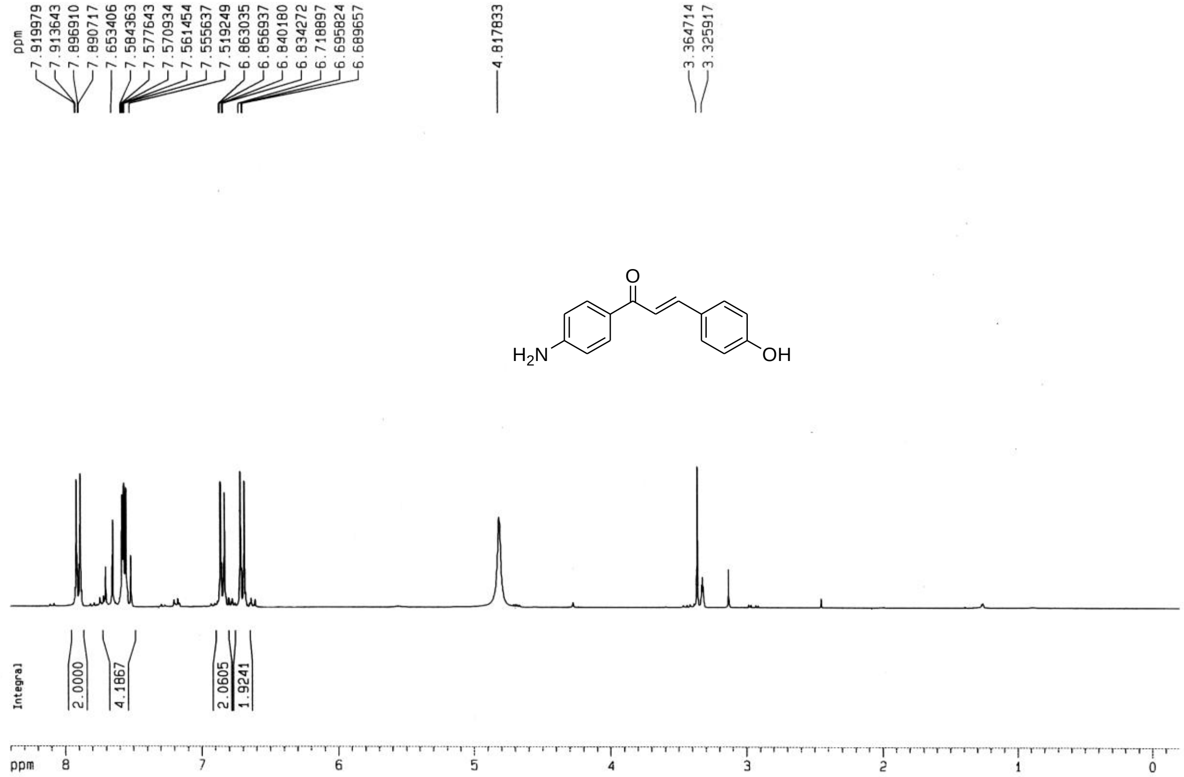

Spectra 2. ^{13}C-NMR of 4'-amino-4-hydroxychalcone (**1**).

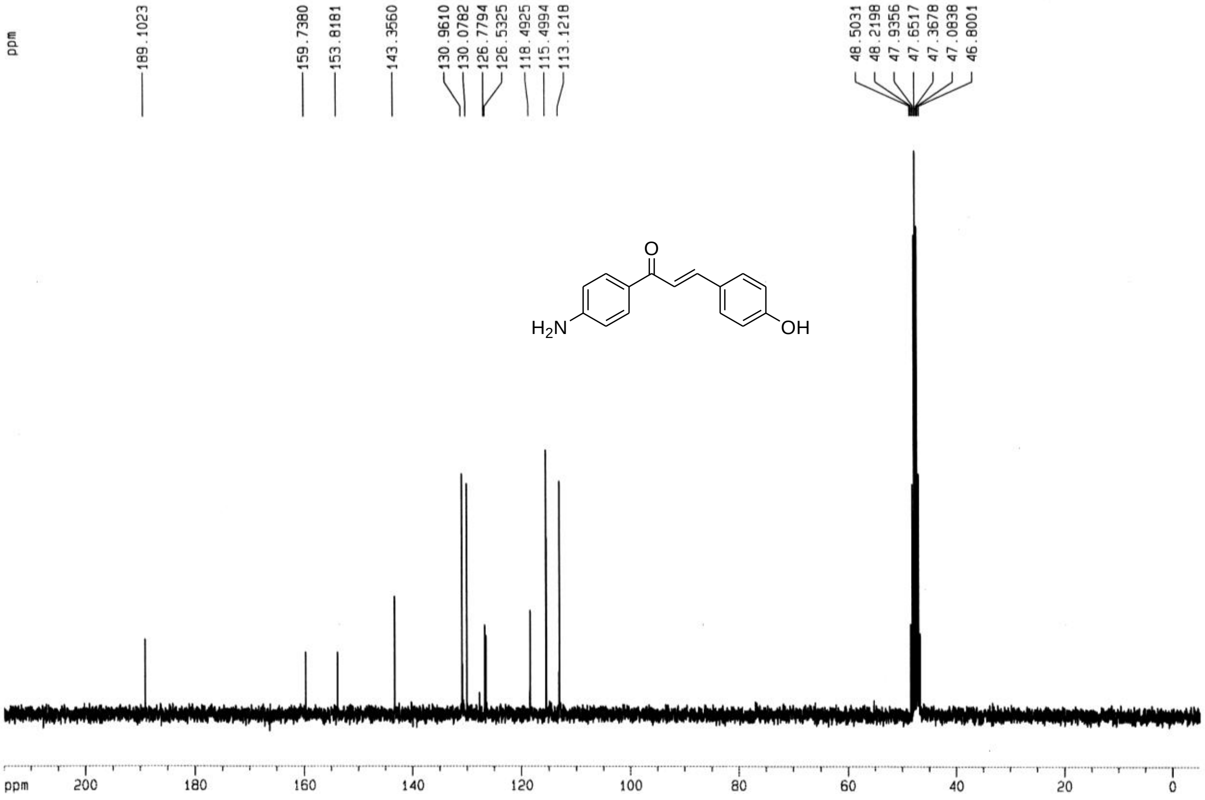

Spectra 3. ^{1}H-NMR of 4'-amino-3,4-dihydroxychalcone (**2**).

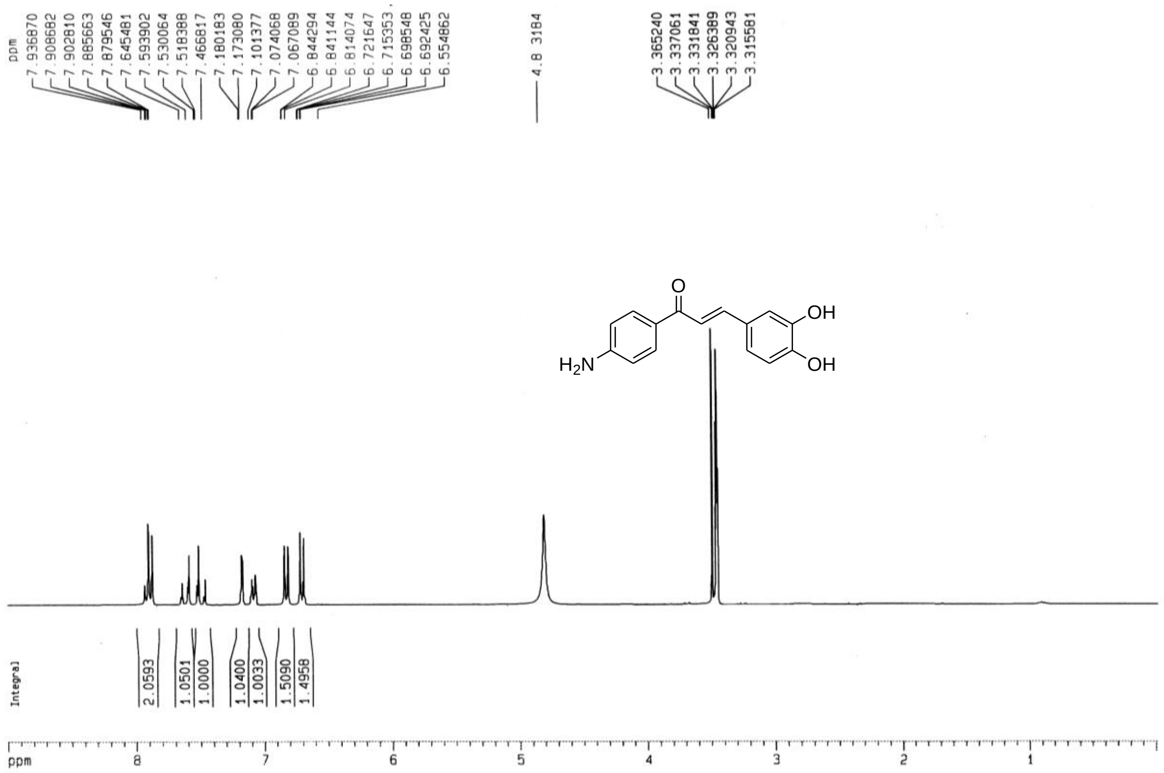

Spectra 4. ^{13}C-NMR of 4'-amino-3,4-dihydroxychalcone (**2**).

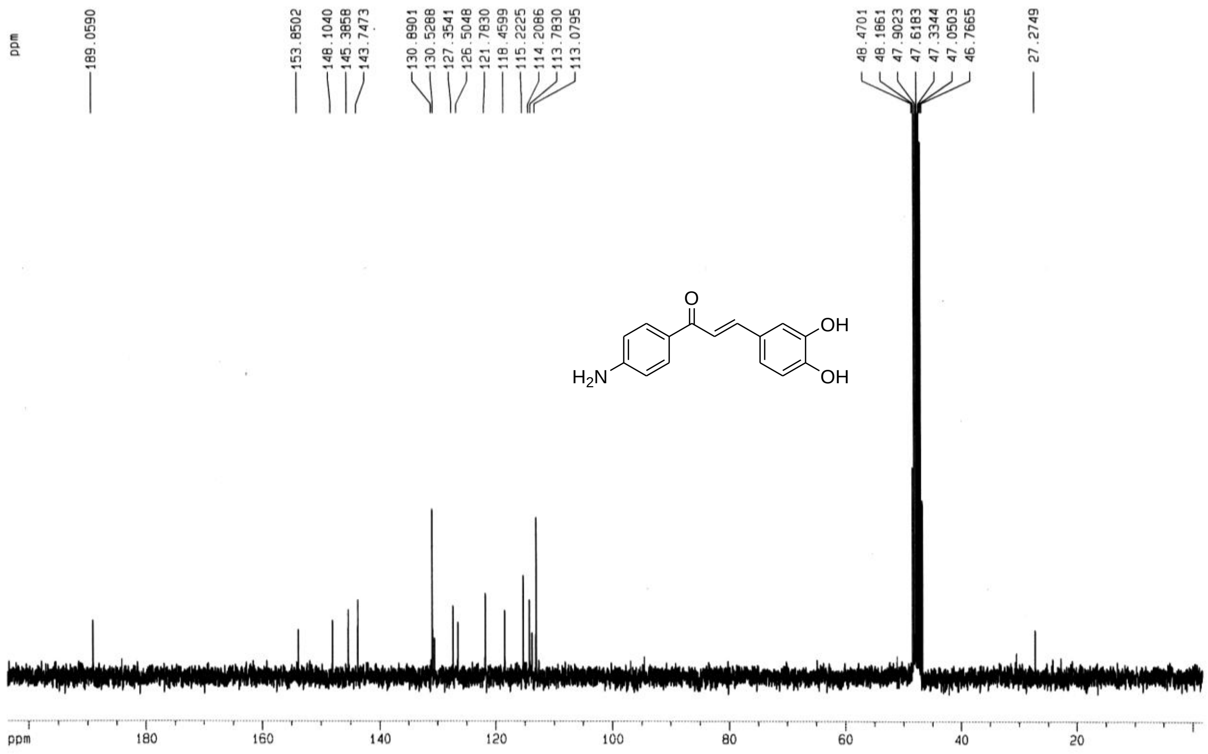

Spectra 5. ^{1}H-NMR of 4'-acetamido-4-hydroxychalcone (**3**).

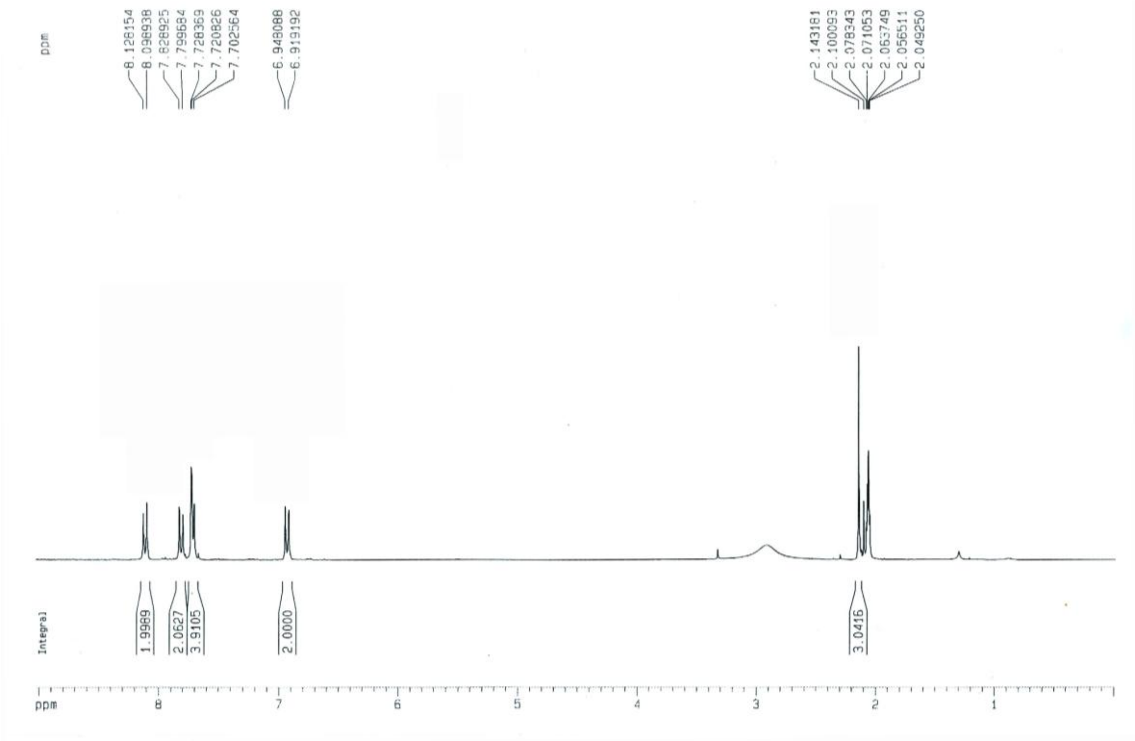

Spectra 6. ^{13}C-NMR of 4'-acetamido-4-hydroxychalcone (**3**).

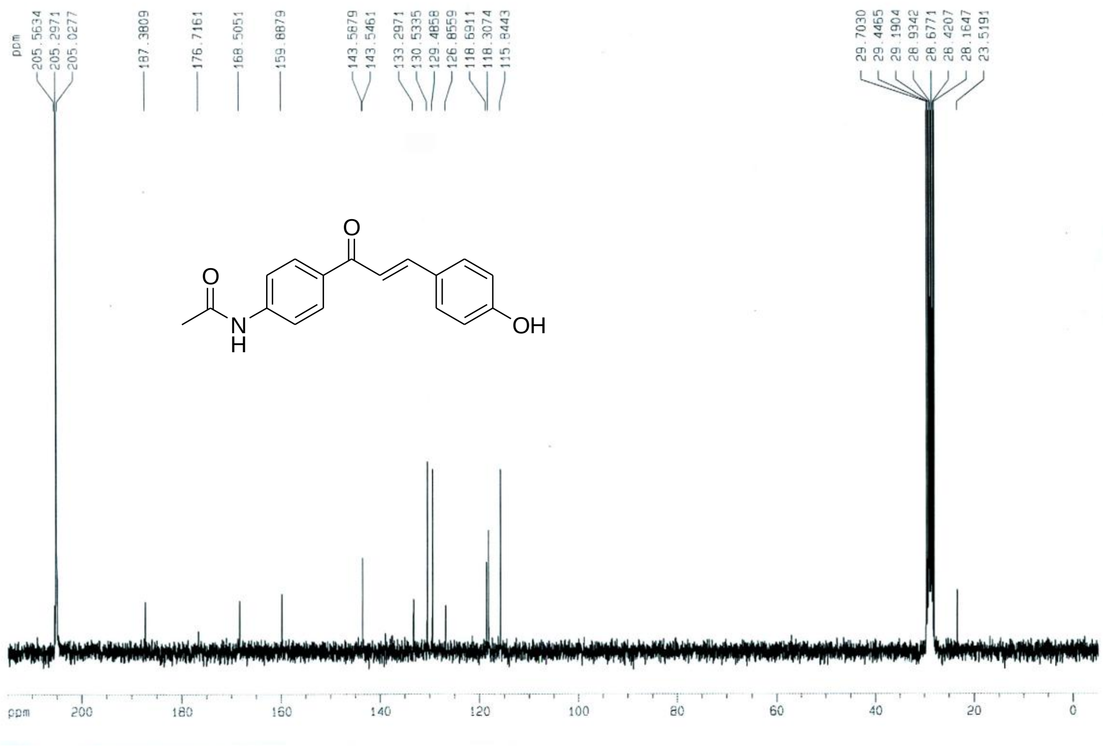

Spectra 7. ^{1}H-NMR of 4'-(*p*-toluenesulfonamide)-4-hydroxychalcone (**4a**).

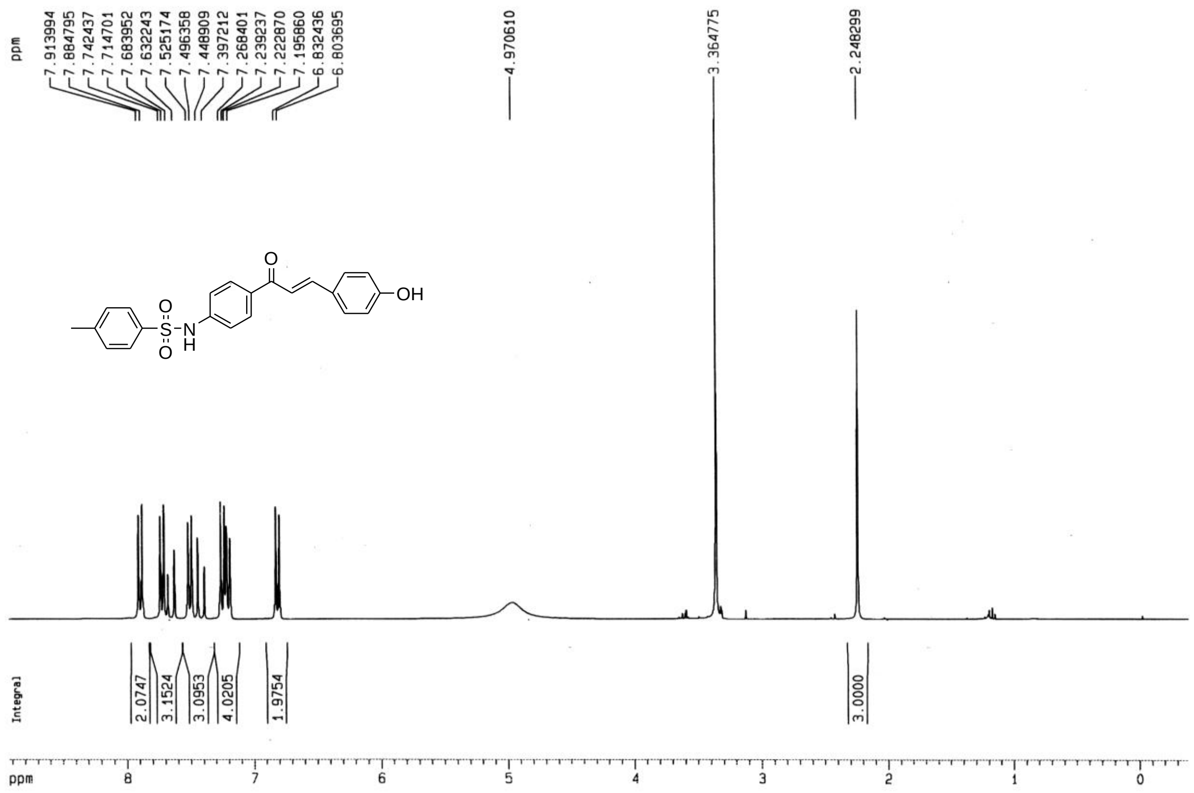

Spectra 8. ^{13}C-NMR of 4'-(*p*-toluenesulfonamide)-4-hydroxychalcone (**4a**).

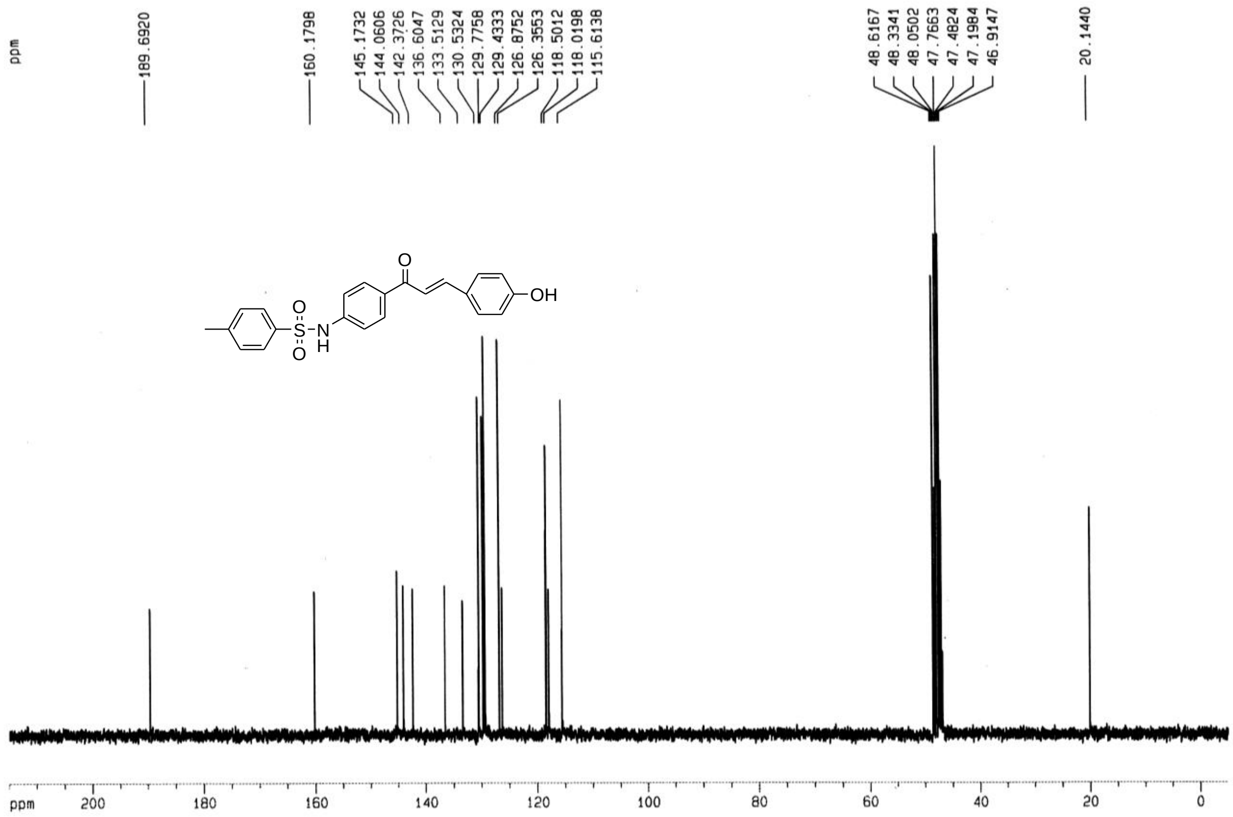

Spectra 9. ^{1}H-NMR of 4'-(4-toluenesulfonamide)-chalcone (**4b**).

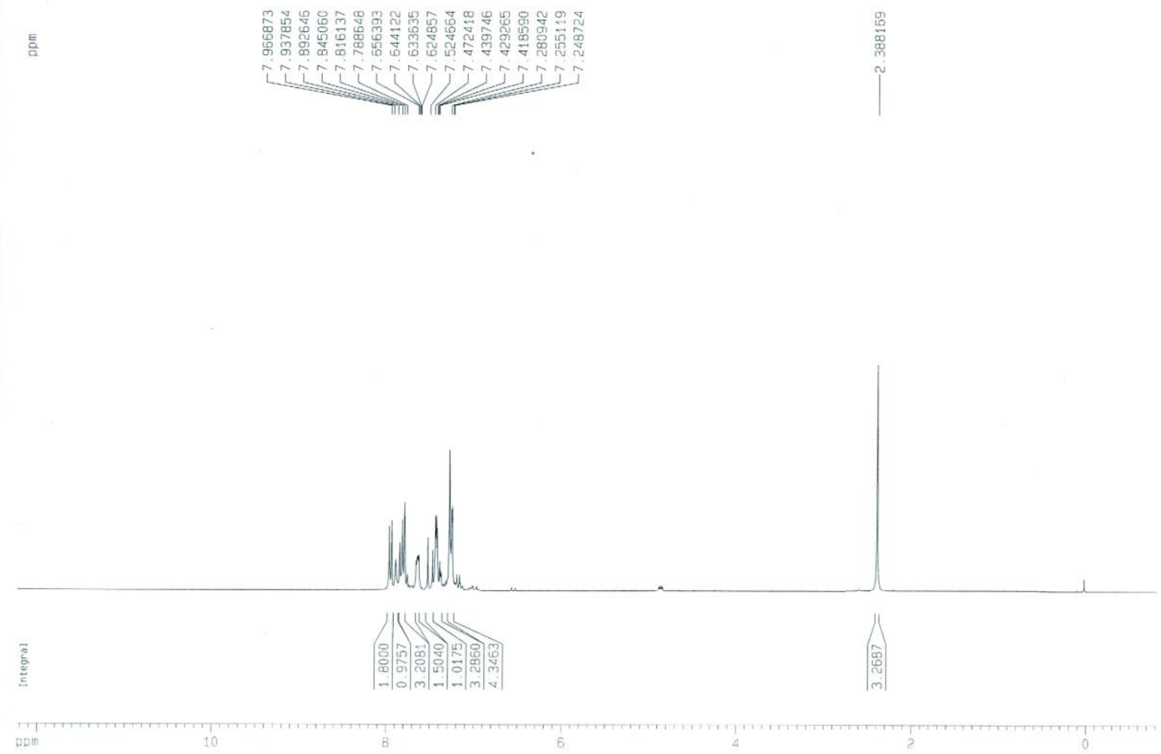

Spectra 10. ^{13}C-NMR of 4'-(4-toluenesulfonamide)-chalcone (**4b**).

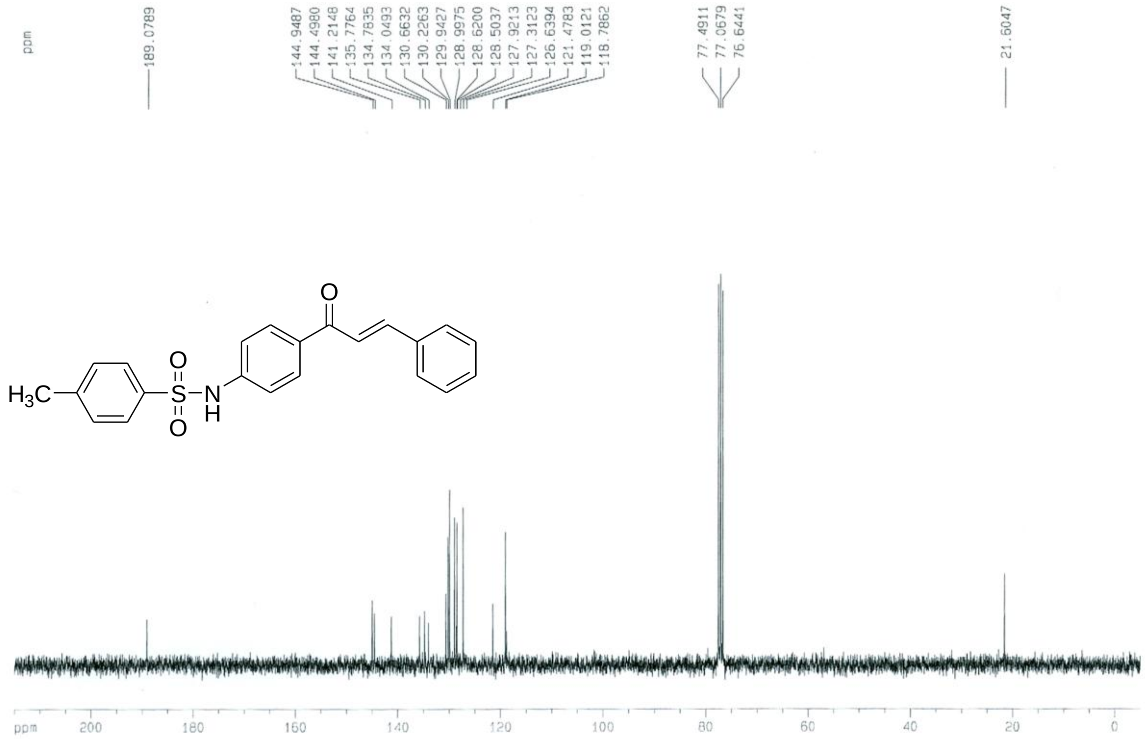

Spectra 11. ^{1}H-NMR of 4'-(benzensulfonamide)-4-hydroxychalcone (**4c**).

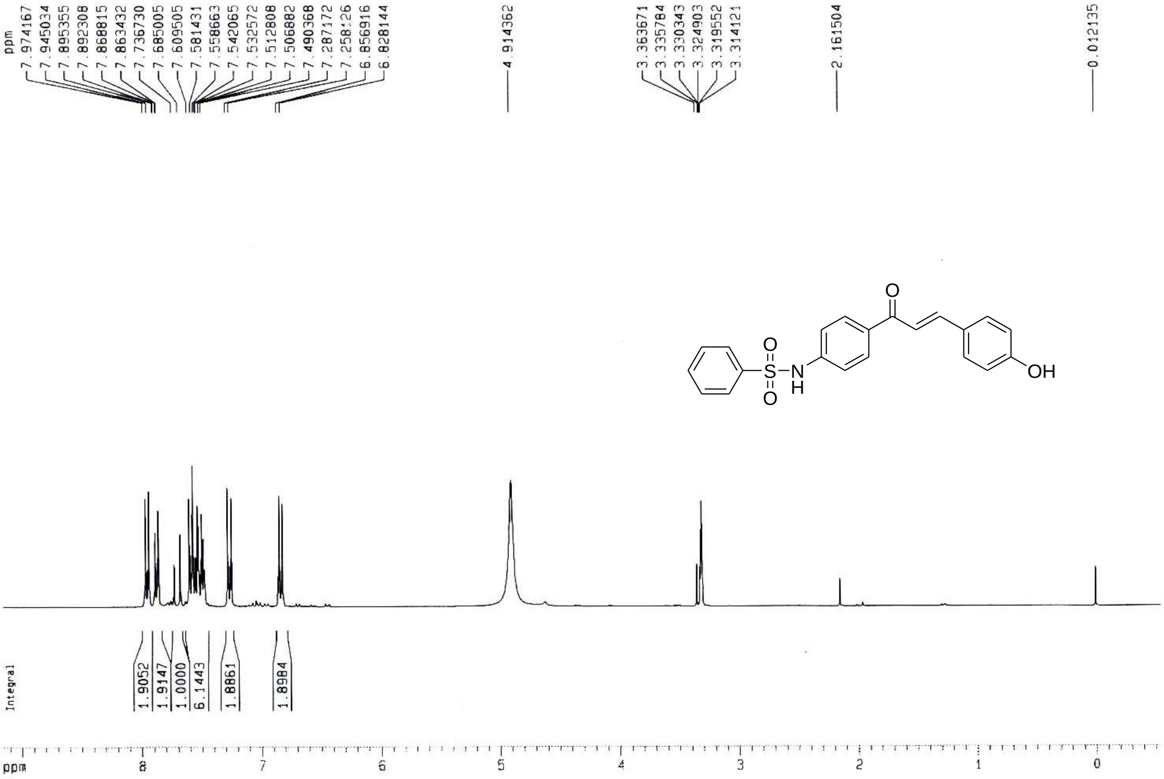

Spectra 12. ^{13}C-NMR of 4'-(benzensulfonamide)-4-hydroxychalcone (**4c**).

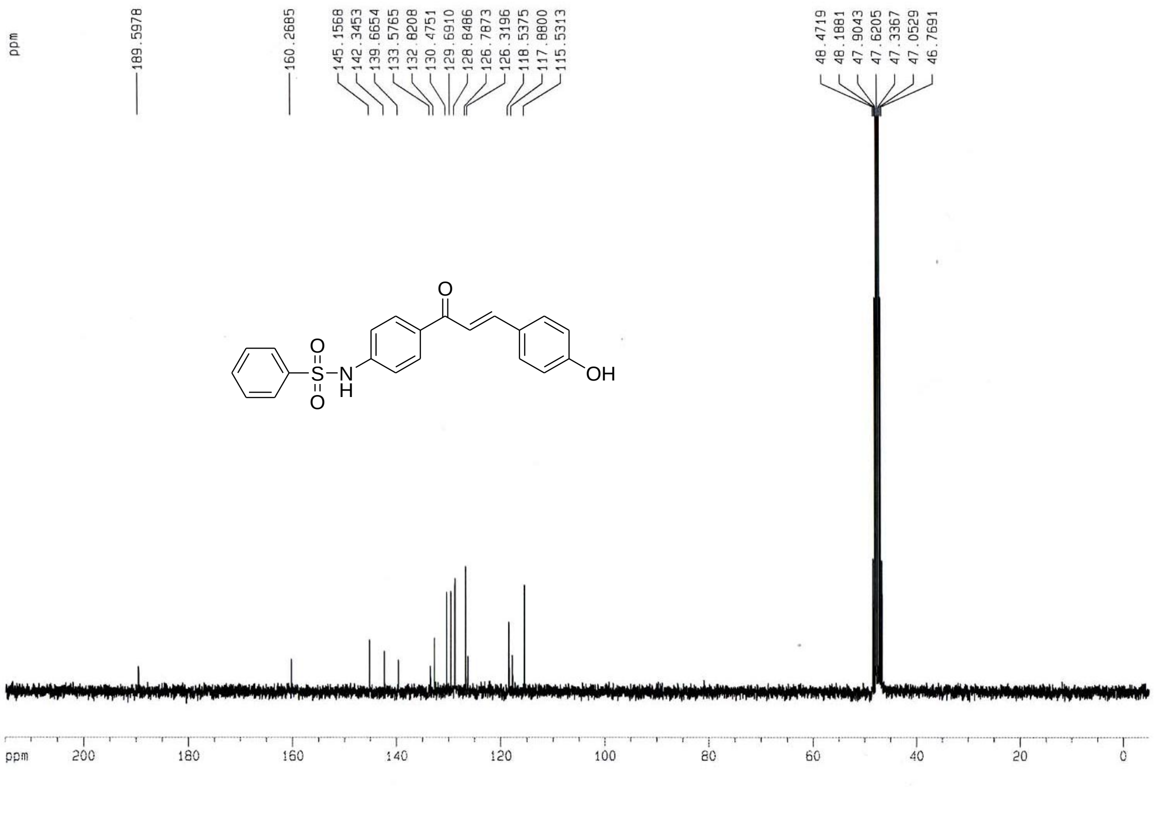

Spectra 13. ^{1}H-NMR of 4'-(4-hydroxybenzensulfonamide)-4-hydroxychalcone (**4d**).

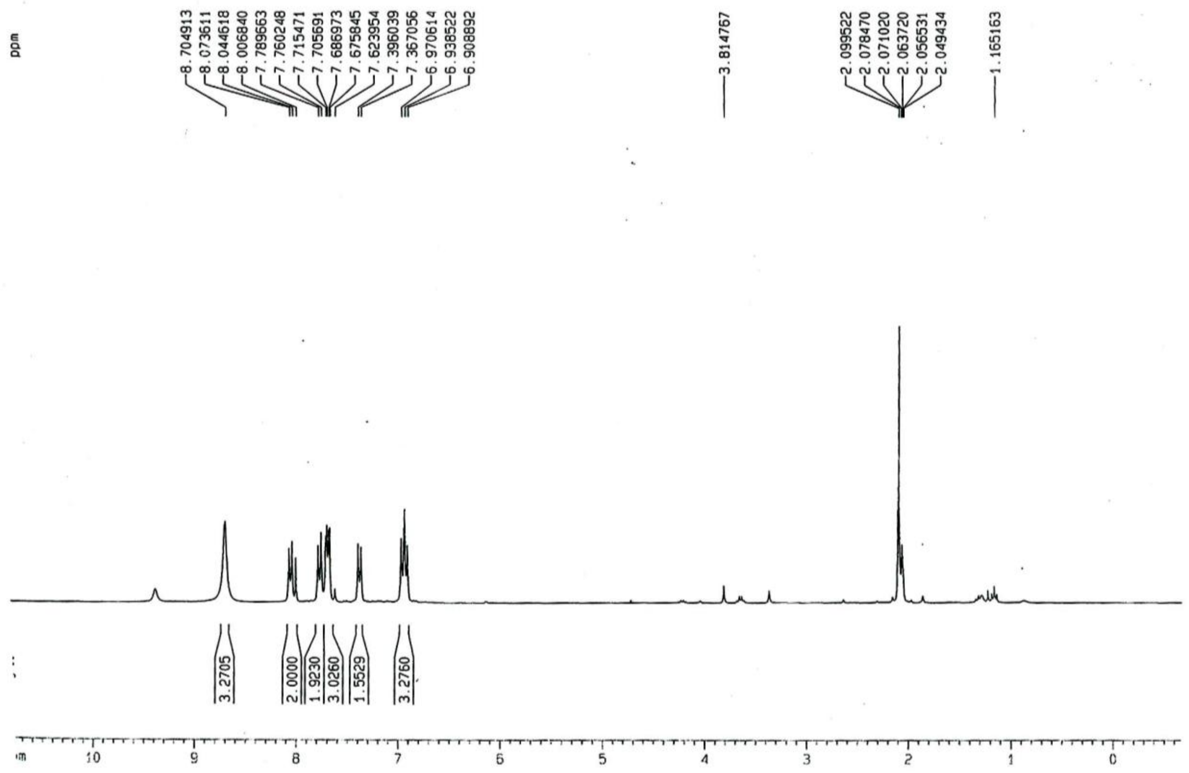

Spectra 14. ^{13}C-NMR of 4'-(4-hydroxybenzensulfonamide)-4-hydroxychalcone (**4d**).

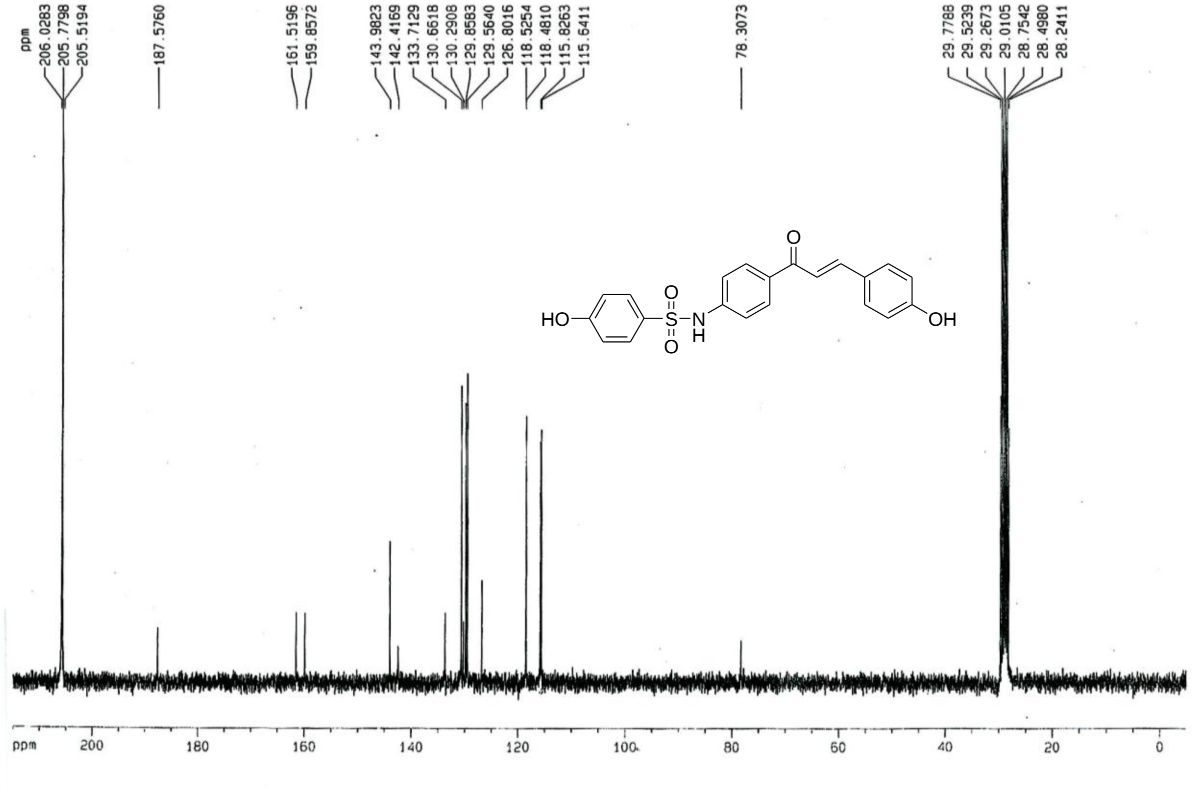

Spectra 15. ^{1}H-NMR of 4'-(4-methoxybezenesulfonamido)-4-hydroxychalcone (**4e**).

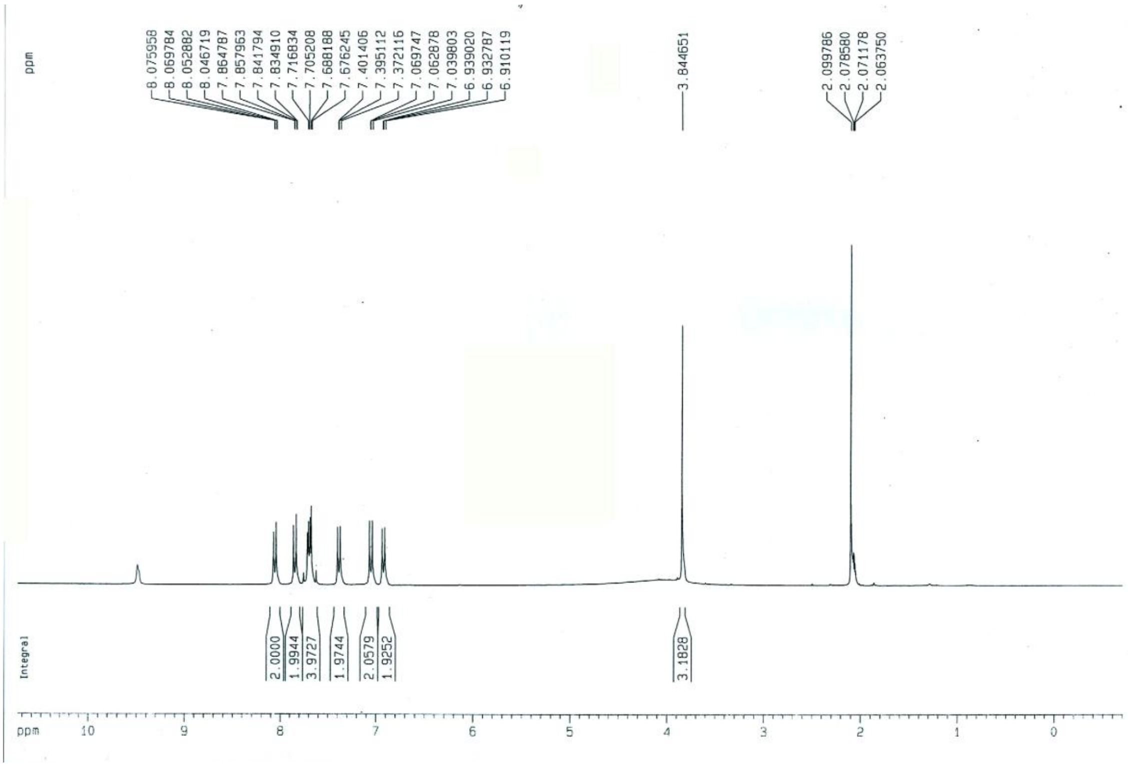

Spectra 16. ^{13}C-NMR of 4'-(4-methoxybezenesulfonamido)-4-hydroxychalcone (**4e**).

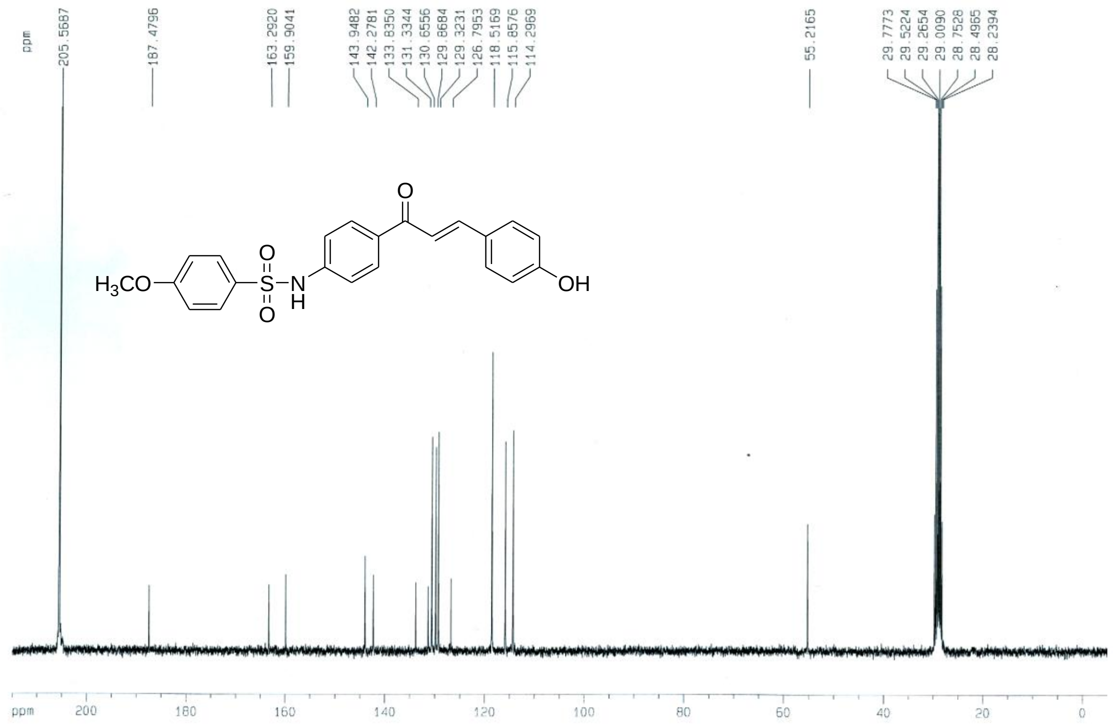

Spectra 17. ^{1}H-NMR of 4'-(4-aminobenzensulfonamide)-4-hydroxychalcone (**4f**).

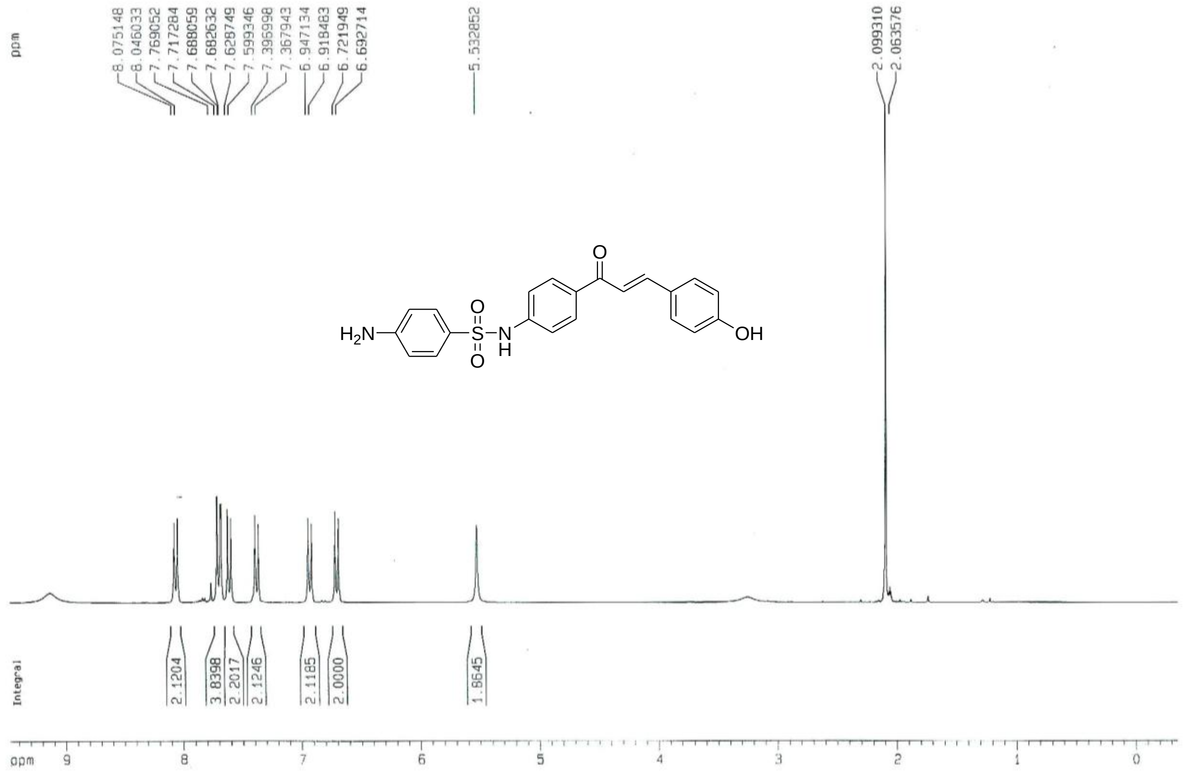

Spectra 18. ^{13}C-NMR of 4'-(4-aminobenzensulfonamide)-4-hydroxychalcone (**4f**).

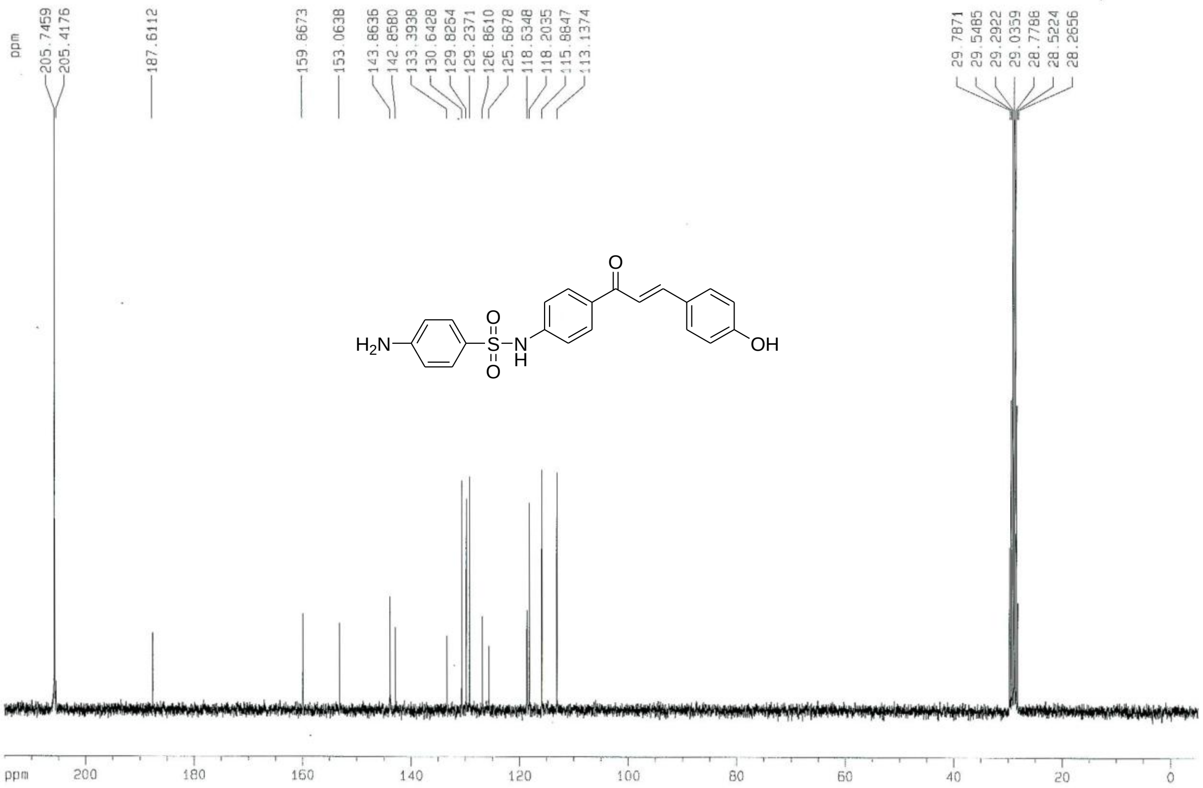

Spectra 19. ^{1}H-NMR of 4'-(4-nitrobenzensulfonamide)-4-hydroxychalcone (**4g**).

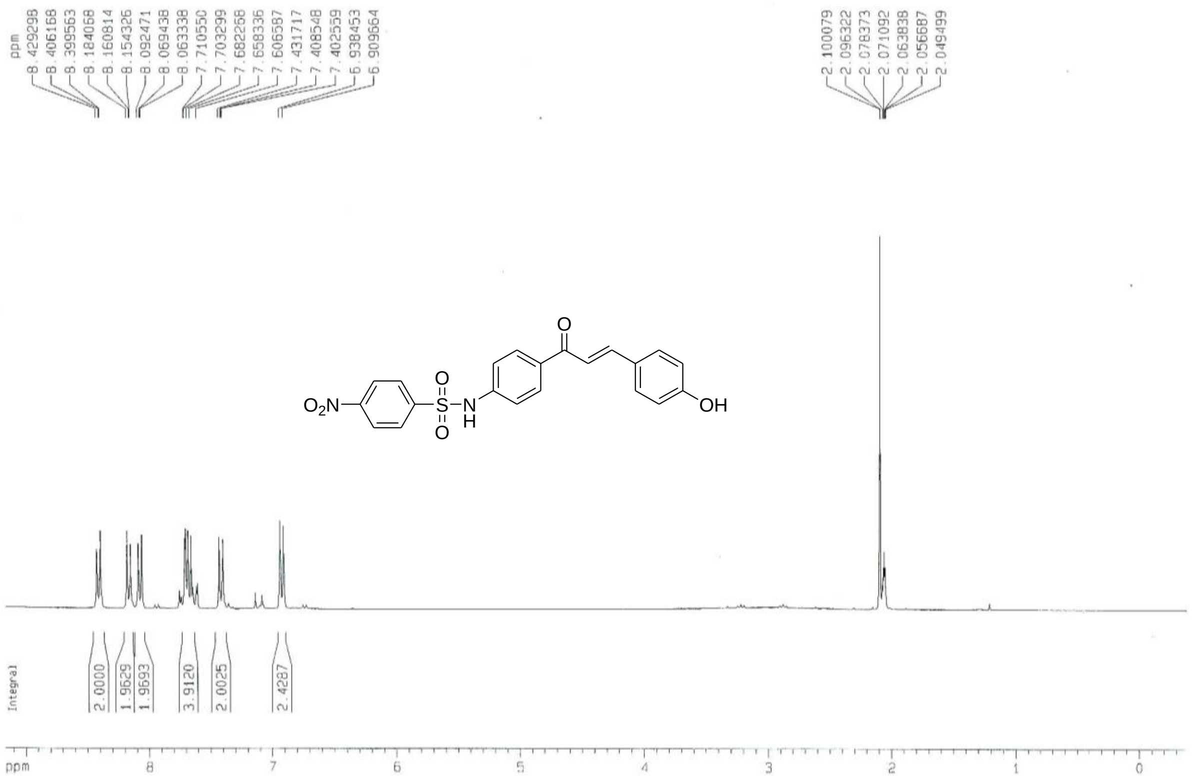

Spectra 20. ^{13}C-NMR of 4'-(4-nitrobenzensulfonamide)-4-hydroxychalcone (**4g**).

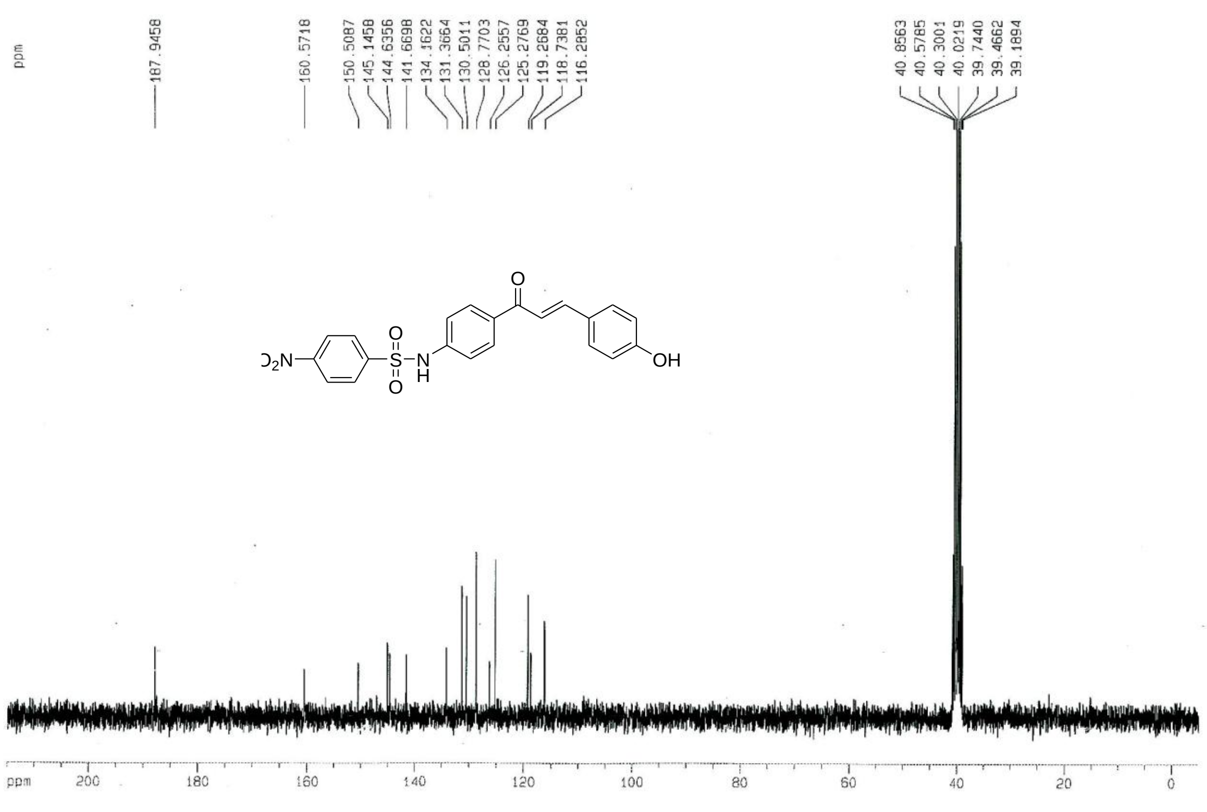

Spectra 21. ^{13}C-NMR of 4'-(4-fluorobenzensulfonamide)-4-hydroxychalcone (**4h**).

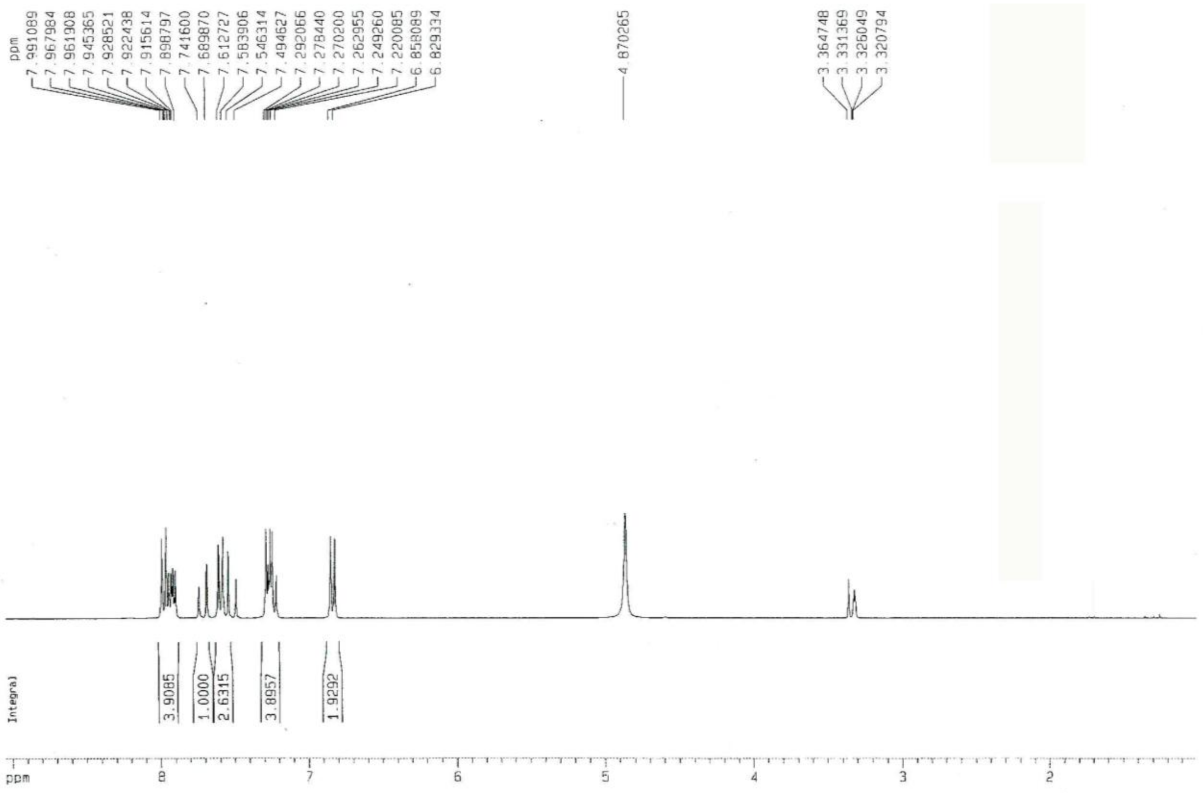

Spectra 22. ^{13}C-NMR of 4'-(4-fluorobenzensulfonamide)-4-hydroxychalcone (**4h**).

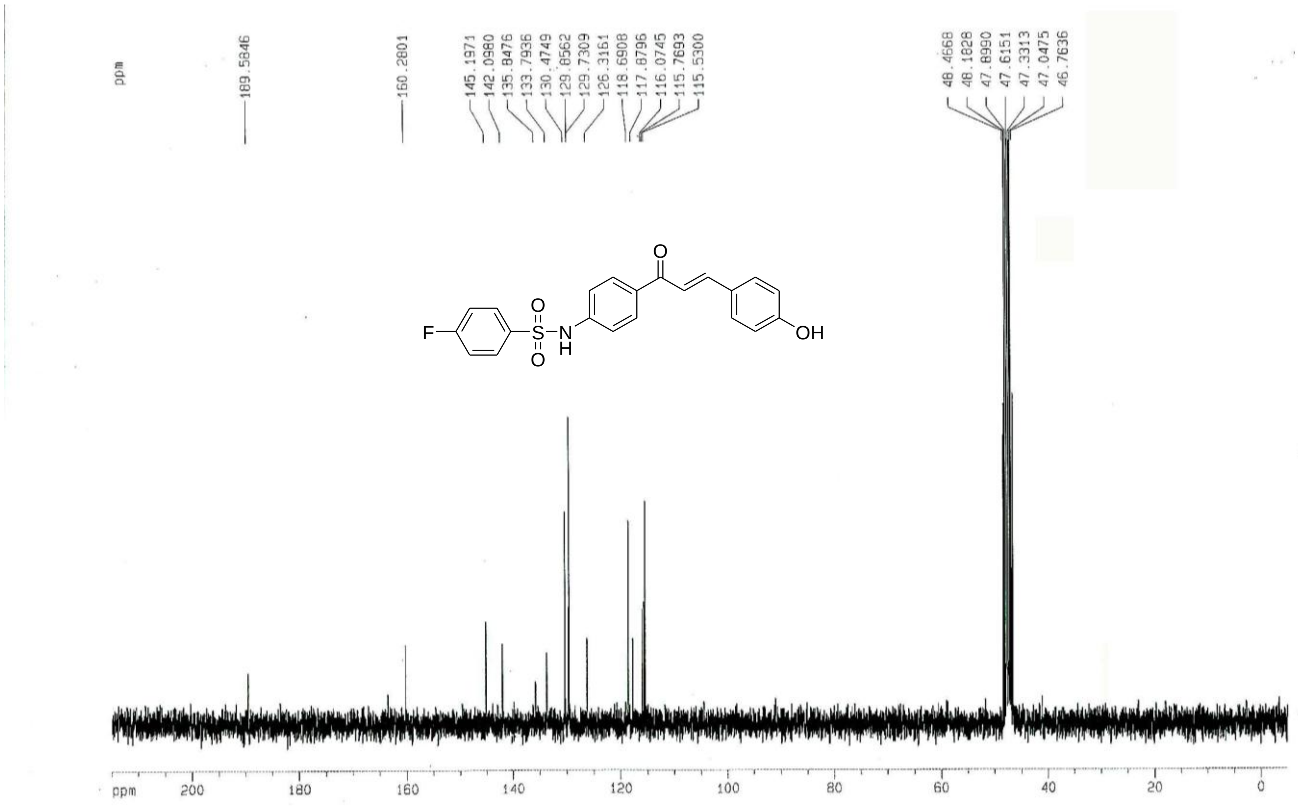

Spectra 23. ^{1}H-NMR of 4'-(*p*-toluenesulfonamide)-3,4-dihydroxychalcone (**5a**).

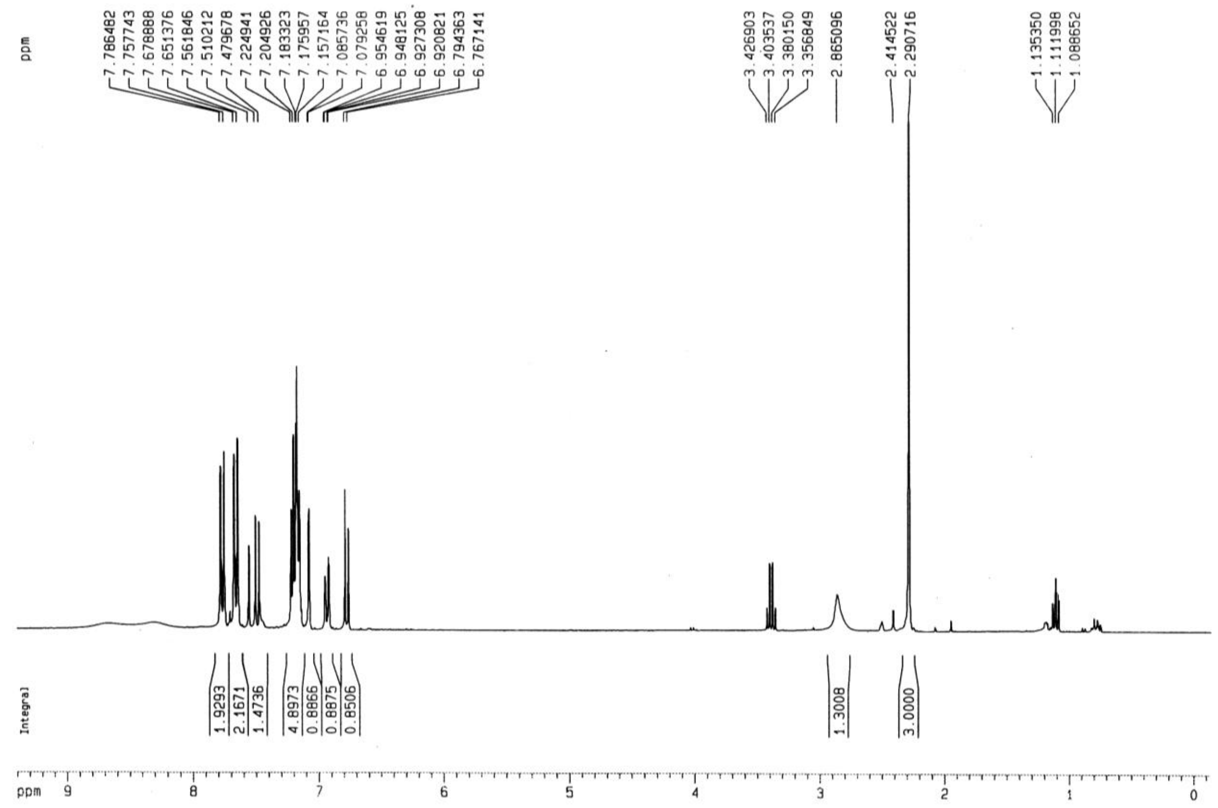

Spectra 24. ^{13}C-NMR of 4'-(*p*-toluenesulfonamide)-3,4-dihydroxychalcone (**5a**).

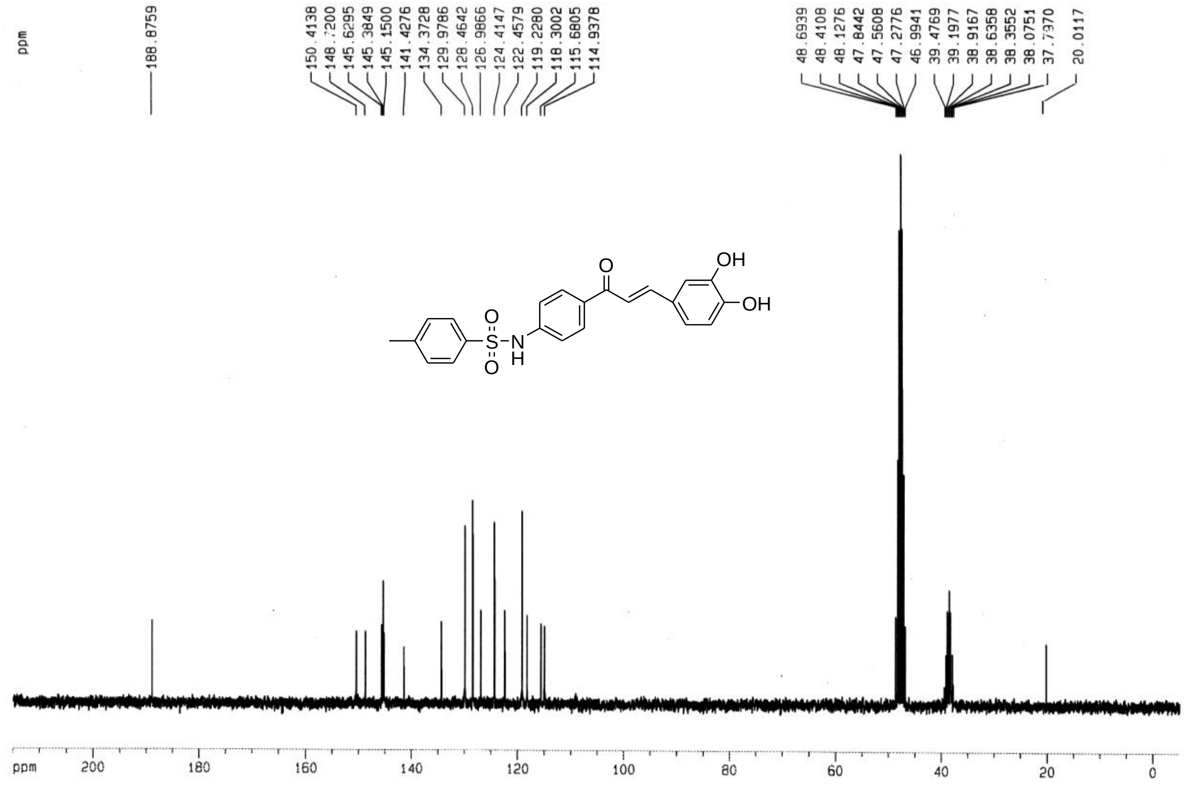

Spectra 25. ^{1}H-NMR of 4'-(benzensulfonamide)-3,4-dihydroxychalcone (**5b**).

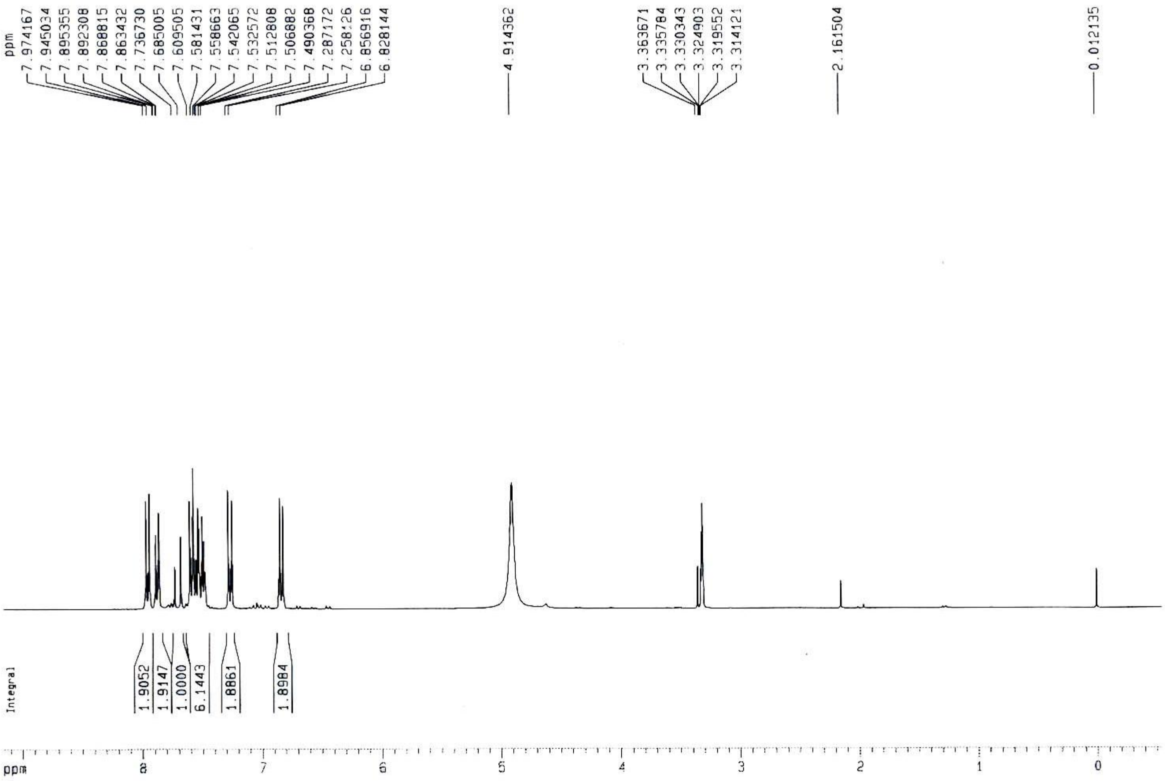

Spectra 26. ^{13}C-NMR of 4'-(benzensulfonamide)-3,4-dihydroxychalcone (**5b**).

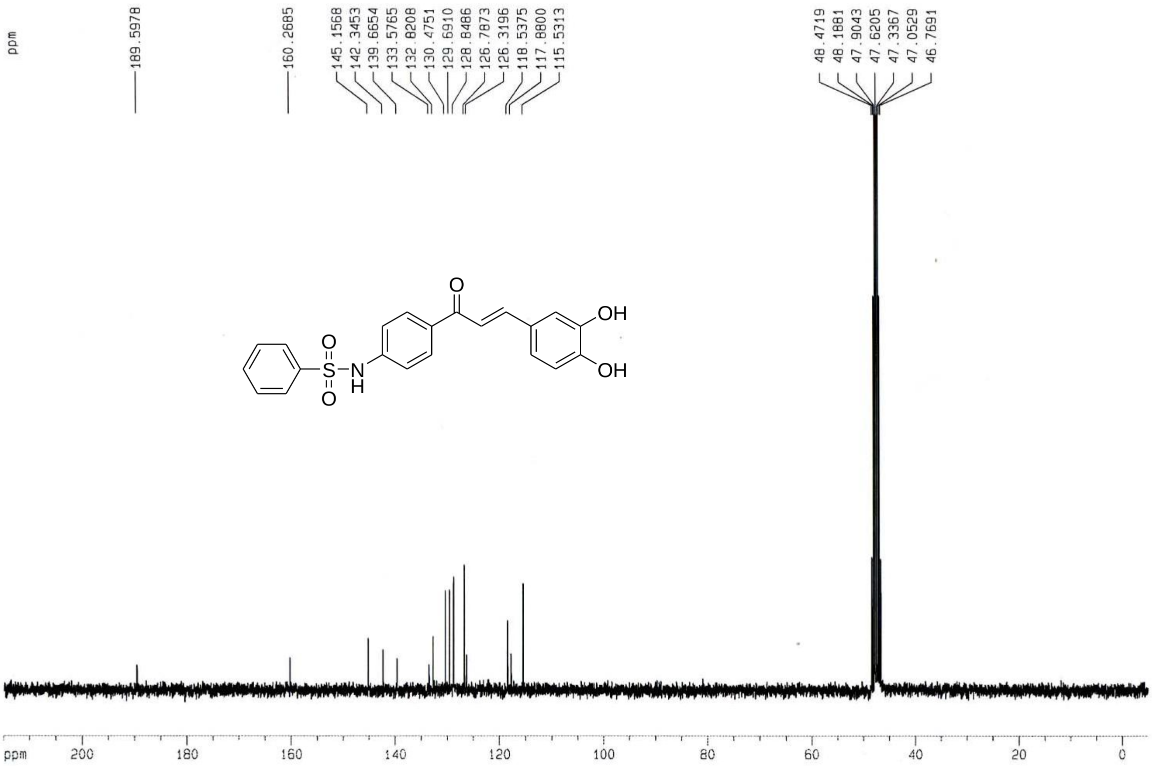

Spectra 27. ^{1}H-NMR of 4'-(4-aminobenzensulfonamide)-3,4-dihydroxychalcone (**5d**).

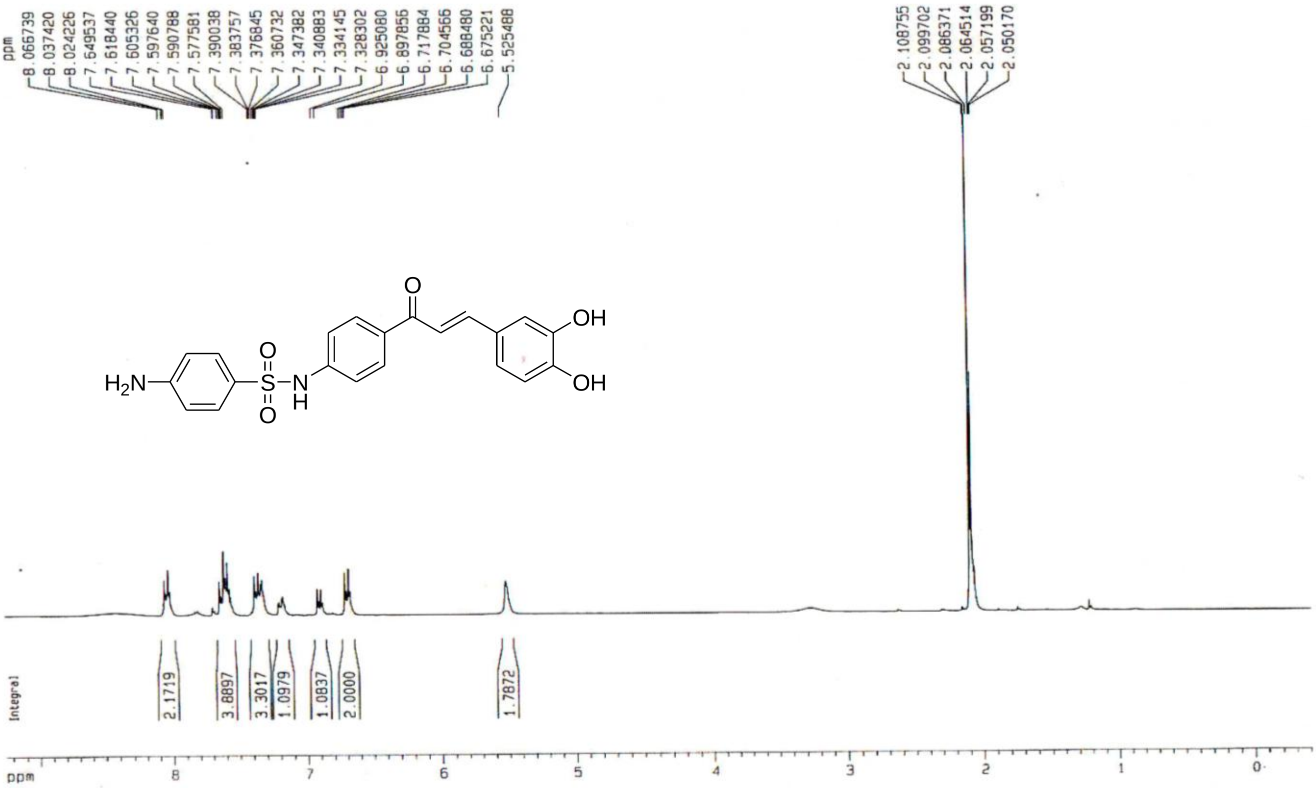

Spectra 28. ^{13}C-NMR of 4'-(4-aminobenzensulfonamide)-3,4-dihydroxychalcone (**5d**).

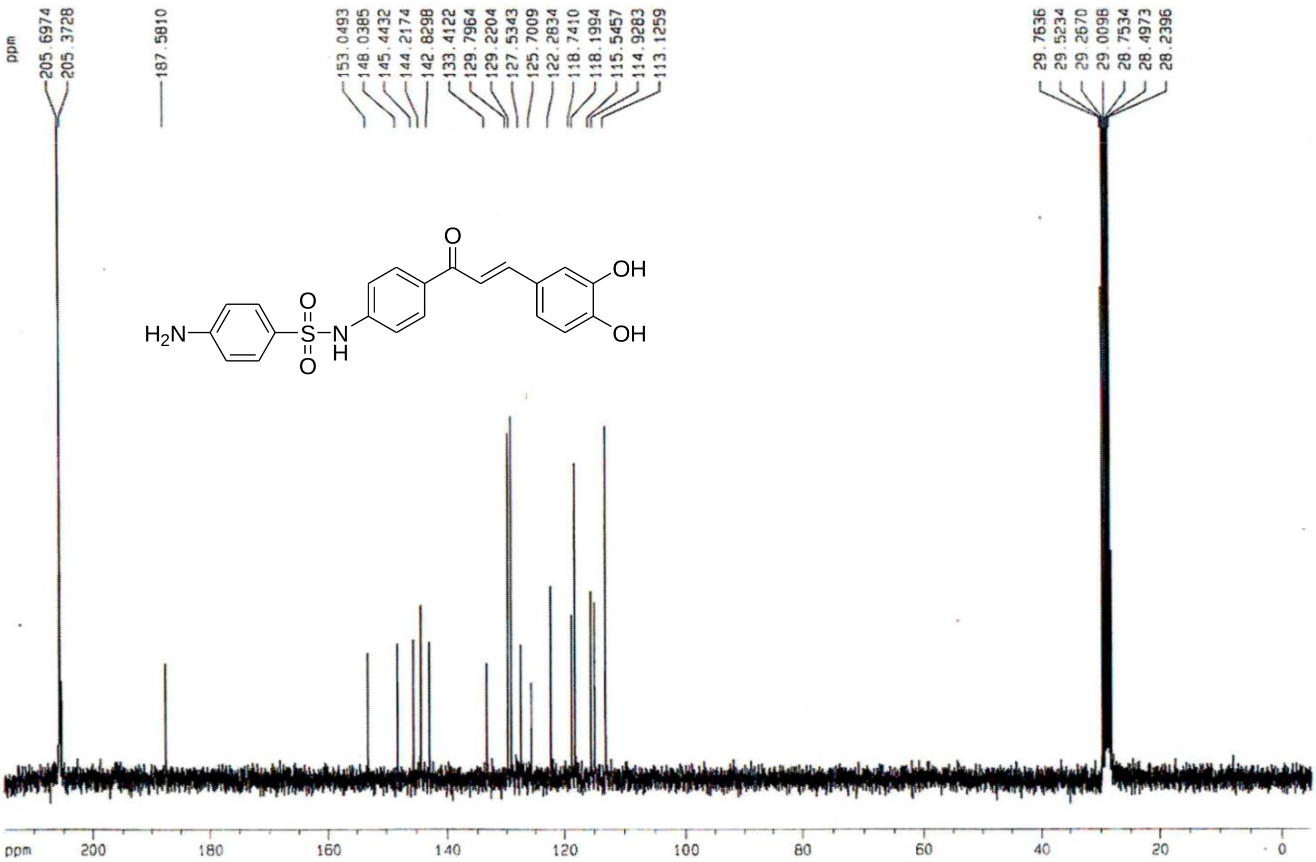

Spectra 29. ^{1}H-NMR of 4'-(4-nitrobenzensulfonamide)-3,4-dihydroxychalcone (**5e**).

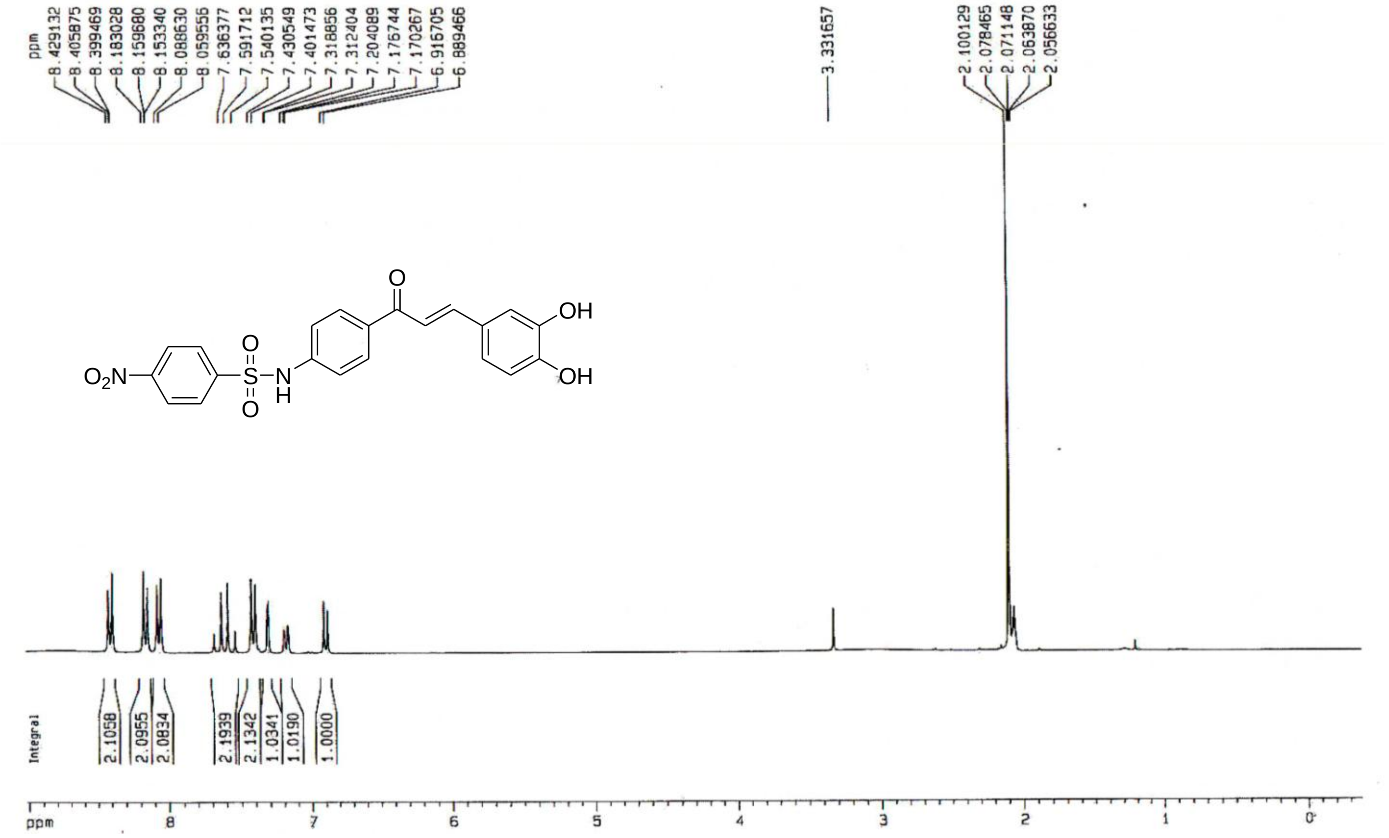

Spectra 30. ^{13}C-NMR of 4'-(4-nitrobenzensulfonamide)-3,4-dihydroxychalcone (**5e**).

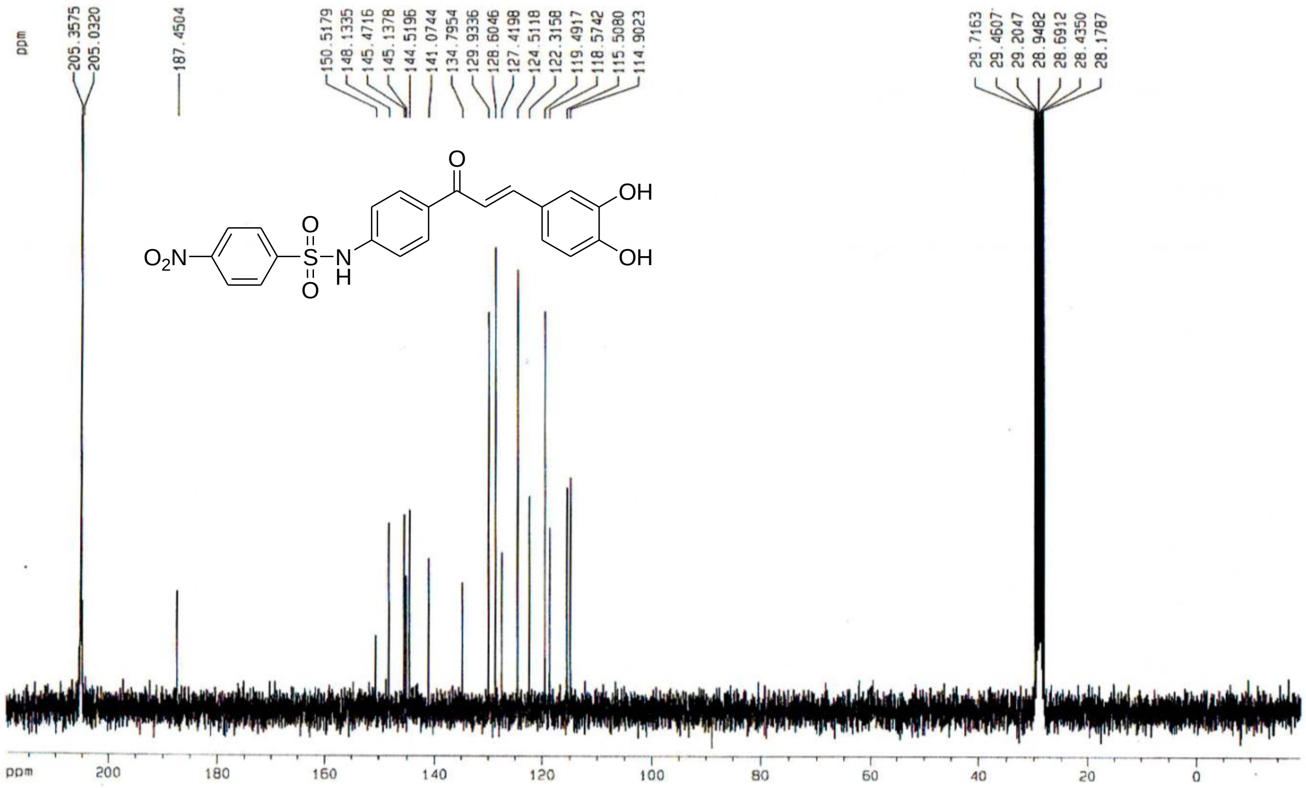

Spectra 31. ^{1}H-NMR of 4'-(4-fluoroenzensulfonamide)-3,4-dihydroxychalcone (**5f**).

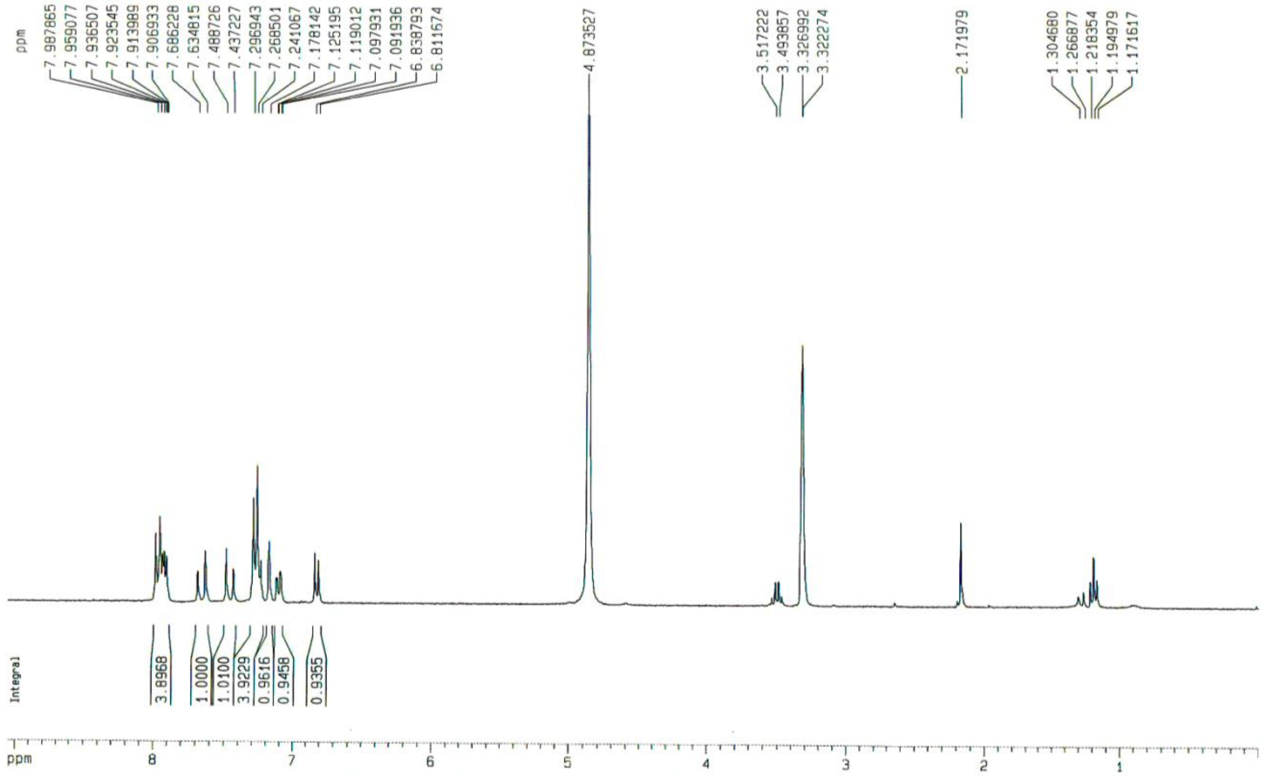

Spectra 32. ^{1}H-NMR of 4'-(*p*-toluenesulfonamide)-2,4-dihydroxychalcone (**6**).

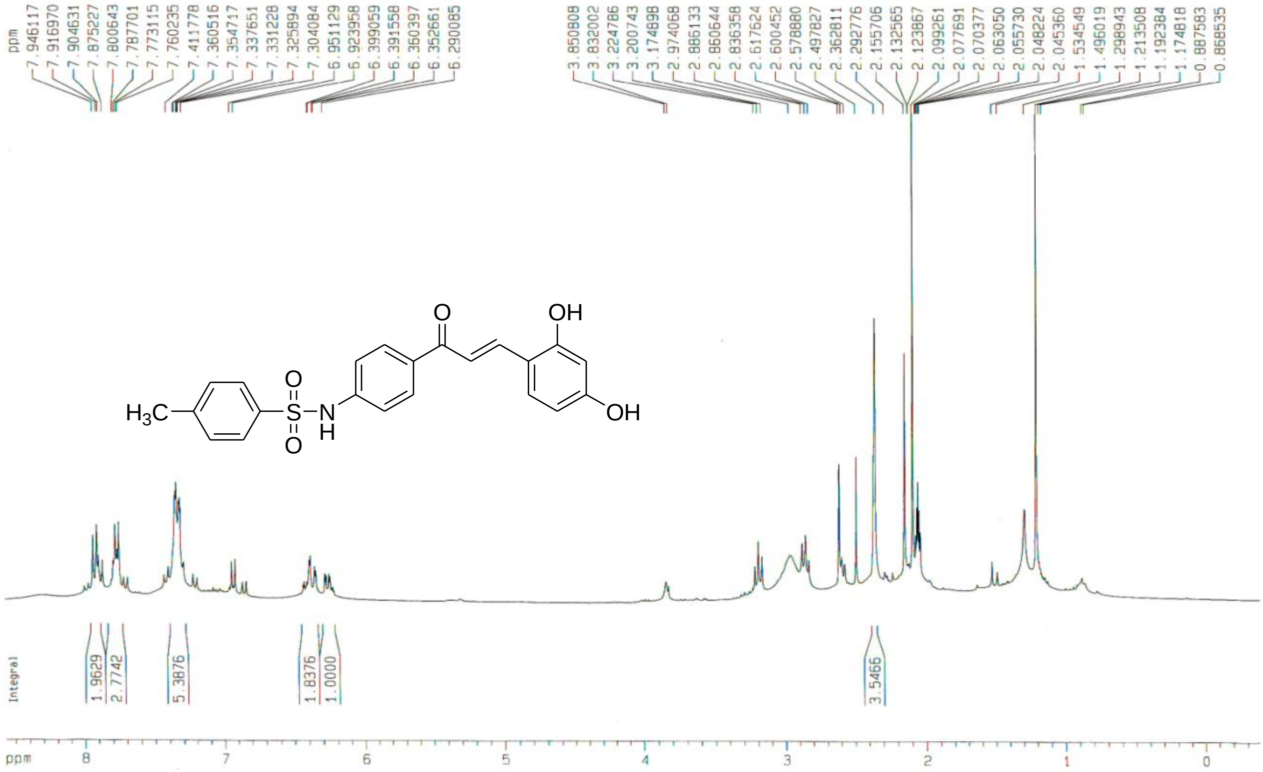

Spectra 33. ^{1}H-NMR of 4'-(*p*-toluenesulfonamide)-2,5-dihydroxychalcone (**7**).

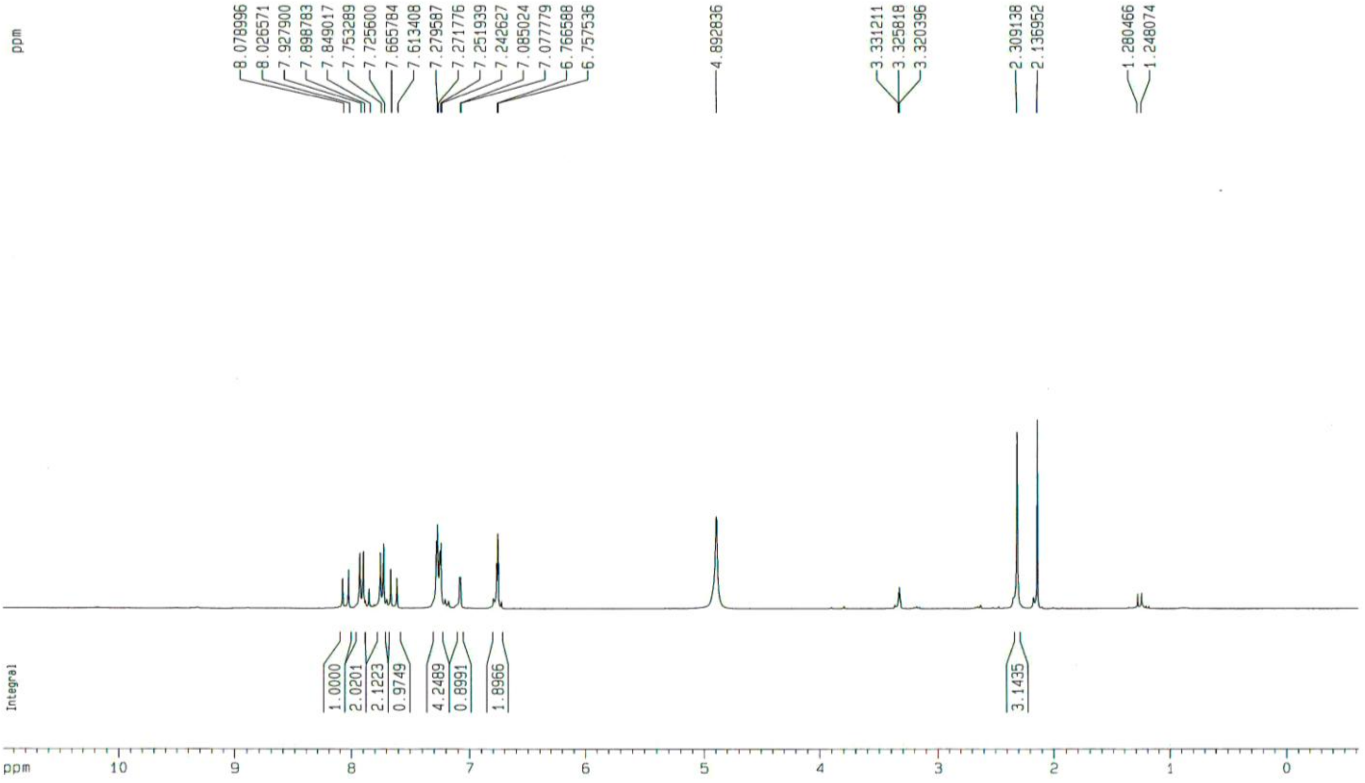

Spectra 34. ^{13}C-NMR of 4'-(*p*-toluenesulfonamide)-2,5-dihydroxychalcone (**7**).

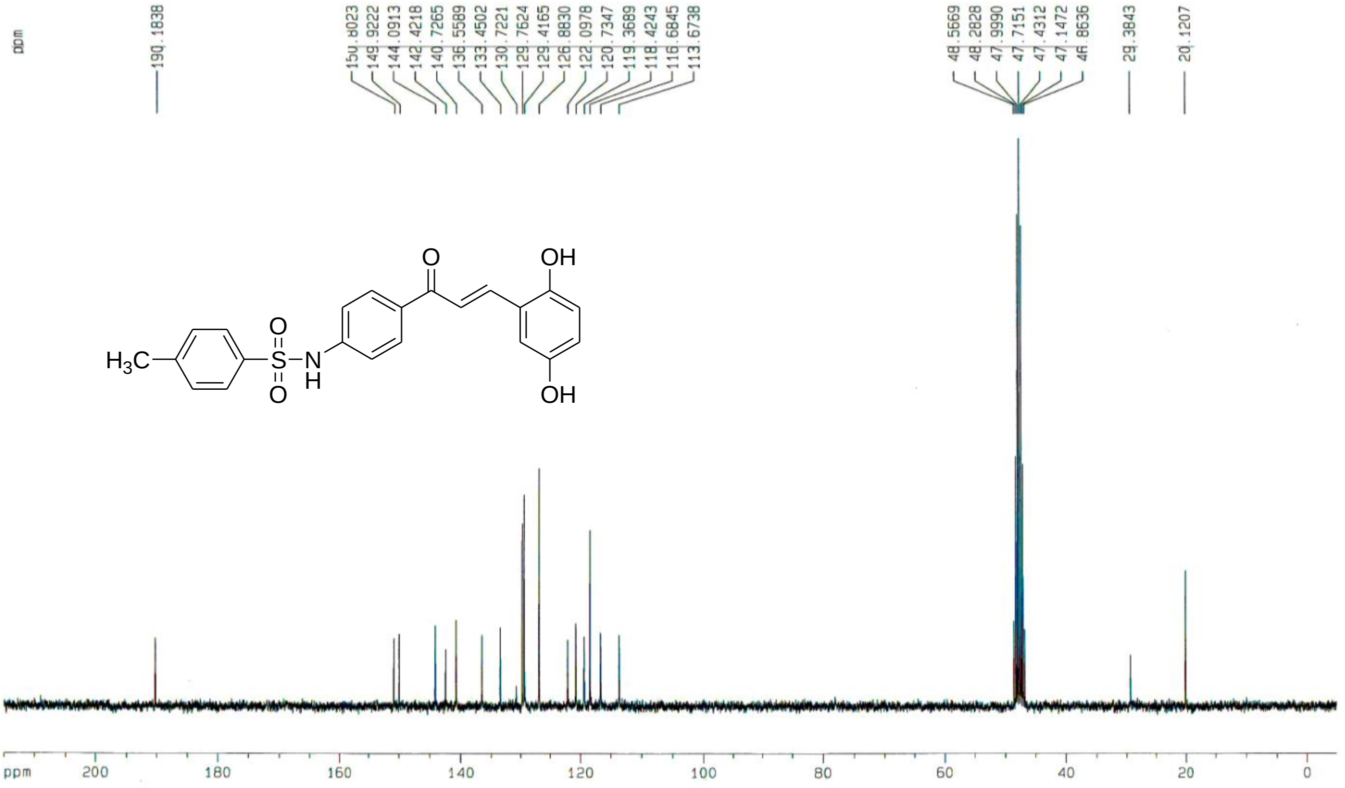

Figure 1. Effect of compounds **1**, **2**, **4a**,**c**, and **5d** on the hydrolytic activity of BACE1. K_m values as a function of the concentrations of **1**, **2**, **4a**,**c**, and **5d**. (Inset) Dependence of the values of V_{max} on the concentration of compounds **1**, **2**, **4a**,**c**, and **5d**.

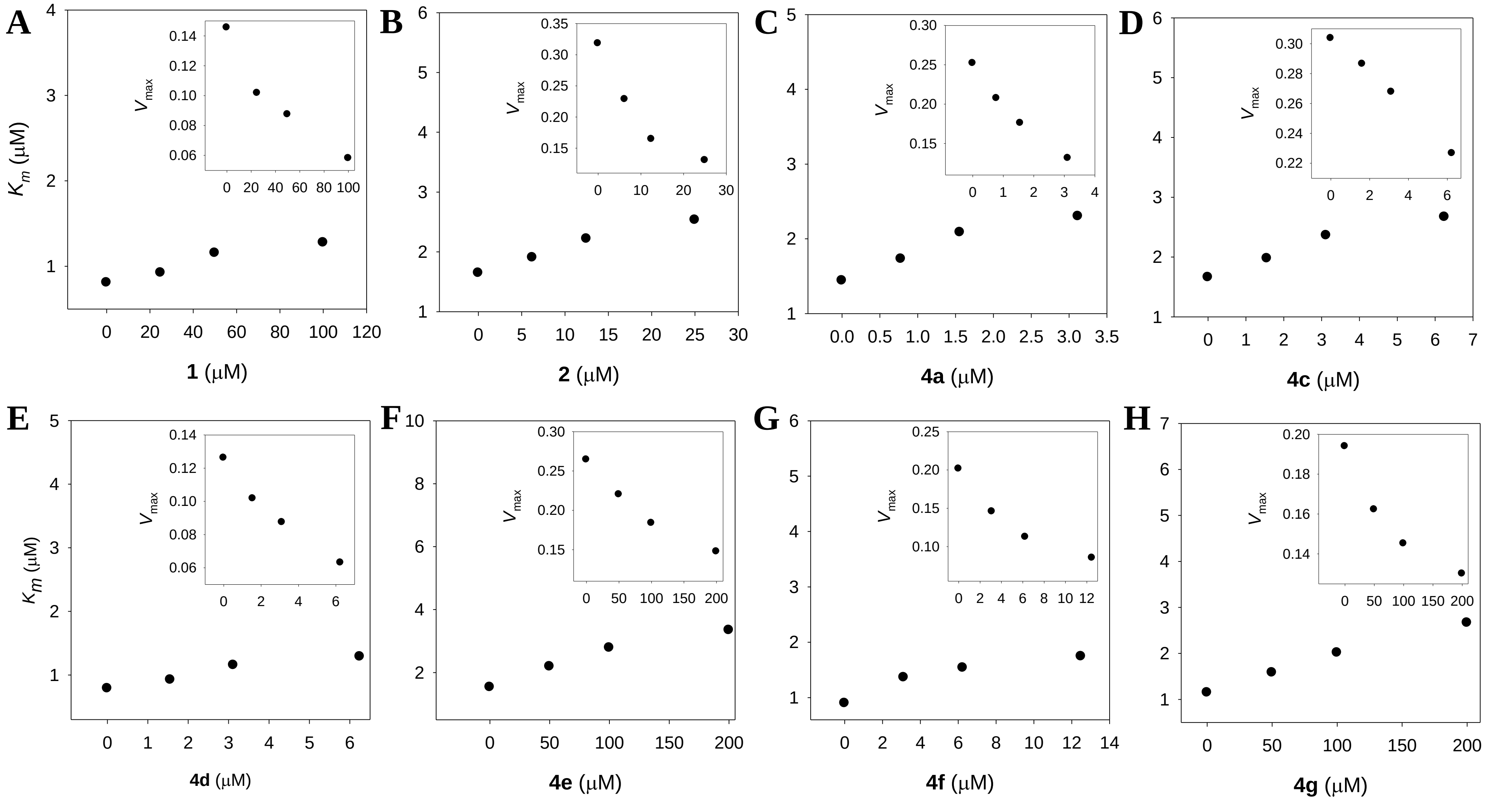

Figure 2. Effect of compounds **1**, **2**, **4a**,**c**, and **5d** on the hydrolytic activity of BACE1. Dixon plots for the inhibition of compounds **1**, **2**, **4a**,**c**, and **5d** on BACE1 catalyzed proteolysis of substrate.

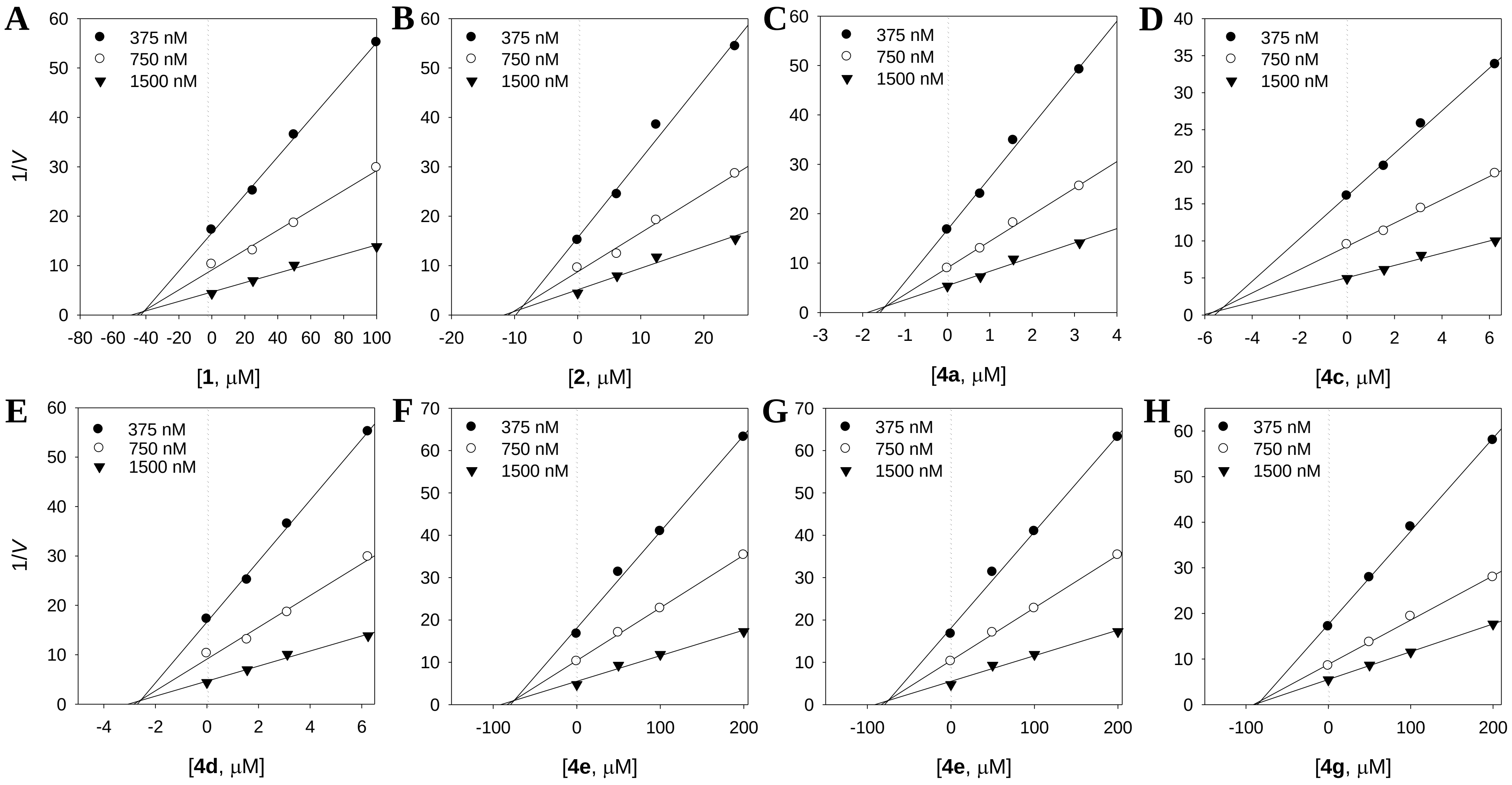

Figure 3. Effect of compounds **5a–d** on the hydrolytic activity of BACE1. K_m values as a function of the concentrations of **5a–d**. (Inset) Dependence of the values of V_{max} on the concentration of compounds **5a–5d**.

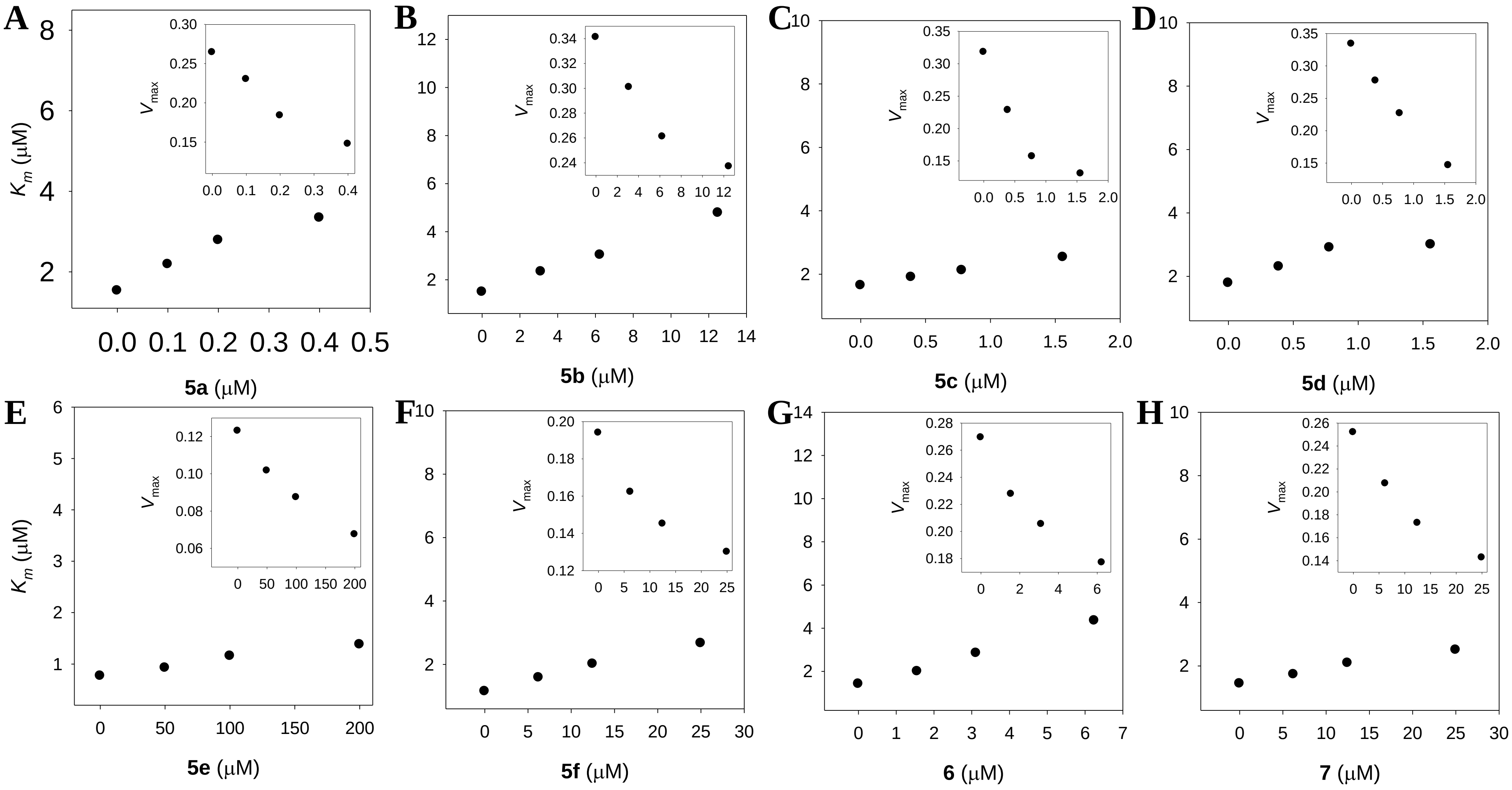

Figure 4. Effect of compounds **5a–d** on the hydrolytic activity of BACE1. Dixon plots for the inhibition of compounds **5a–d** on BACE1 catalyzed proteolysis of substrate.

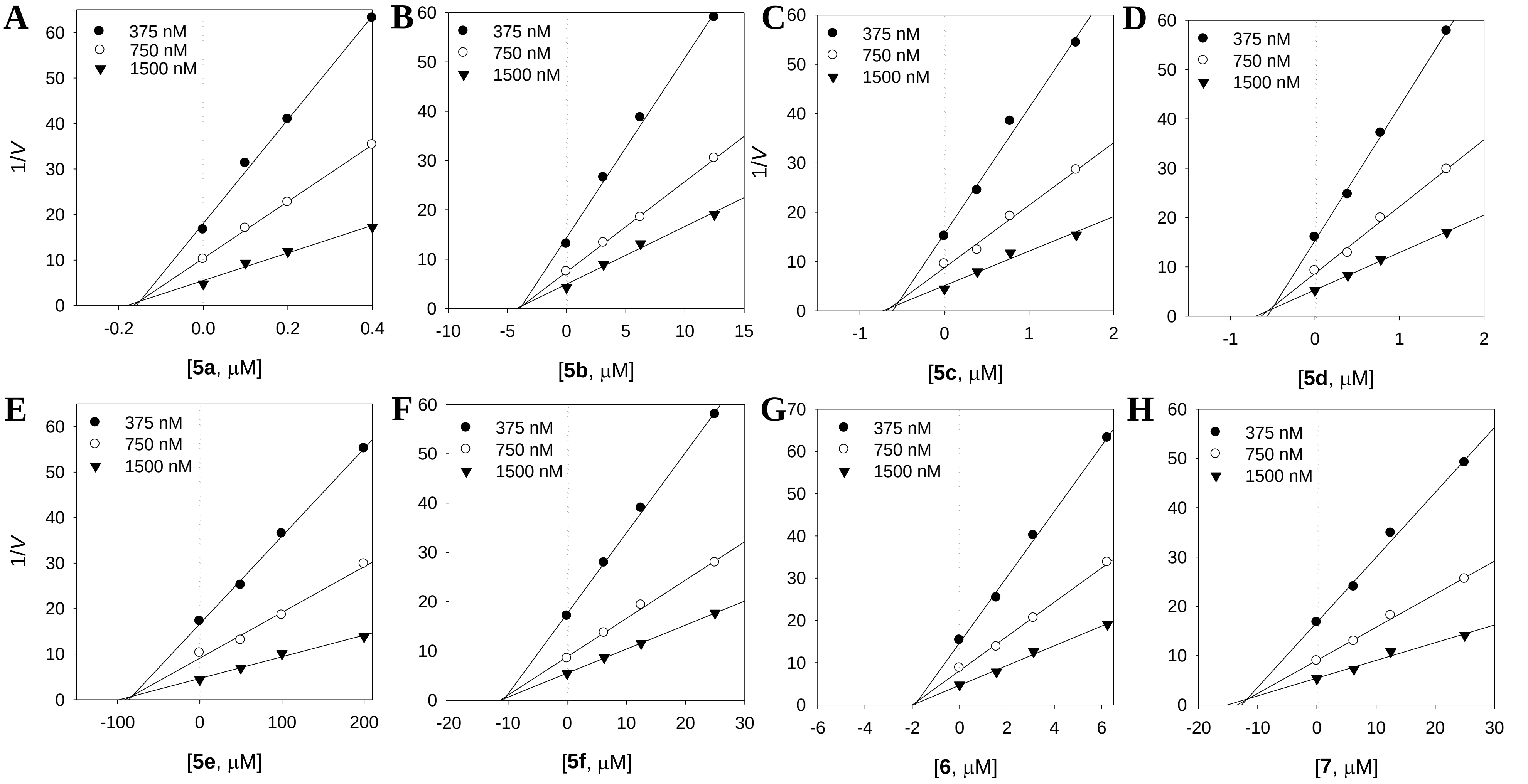

Figure 5. Acetylcholinesterase kinetic data of compounds **4a**,**f**, and **5b**,**d**.

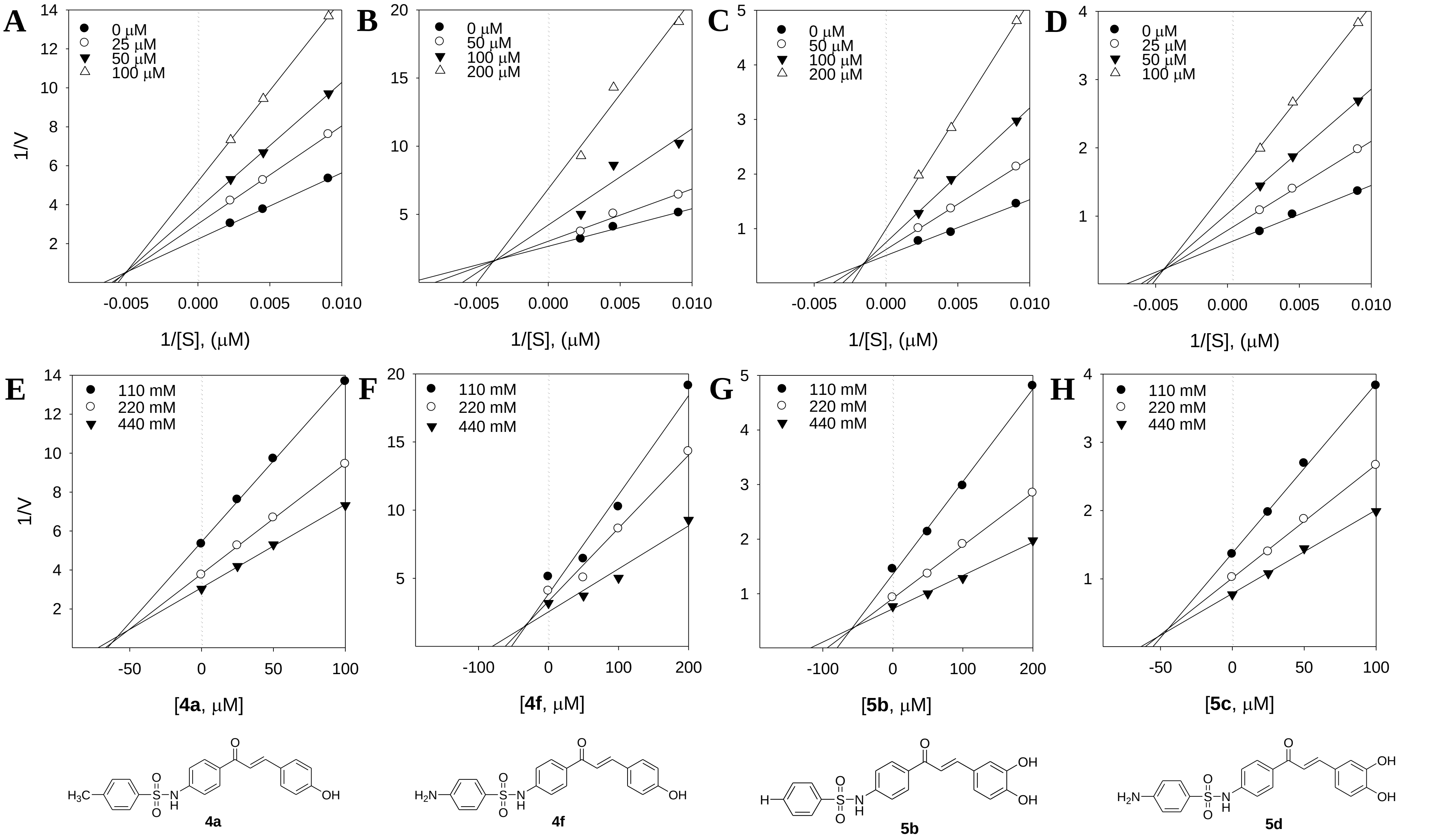

Figure 6. Butyrylcholinesterase kinetic data of compounds **4a,f**, and **5b,d**.

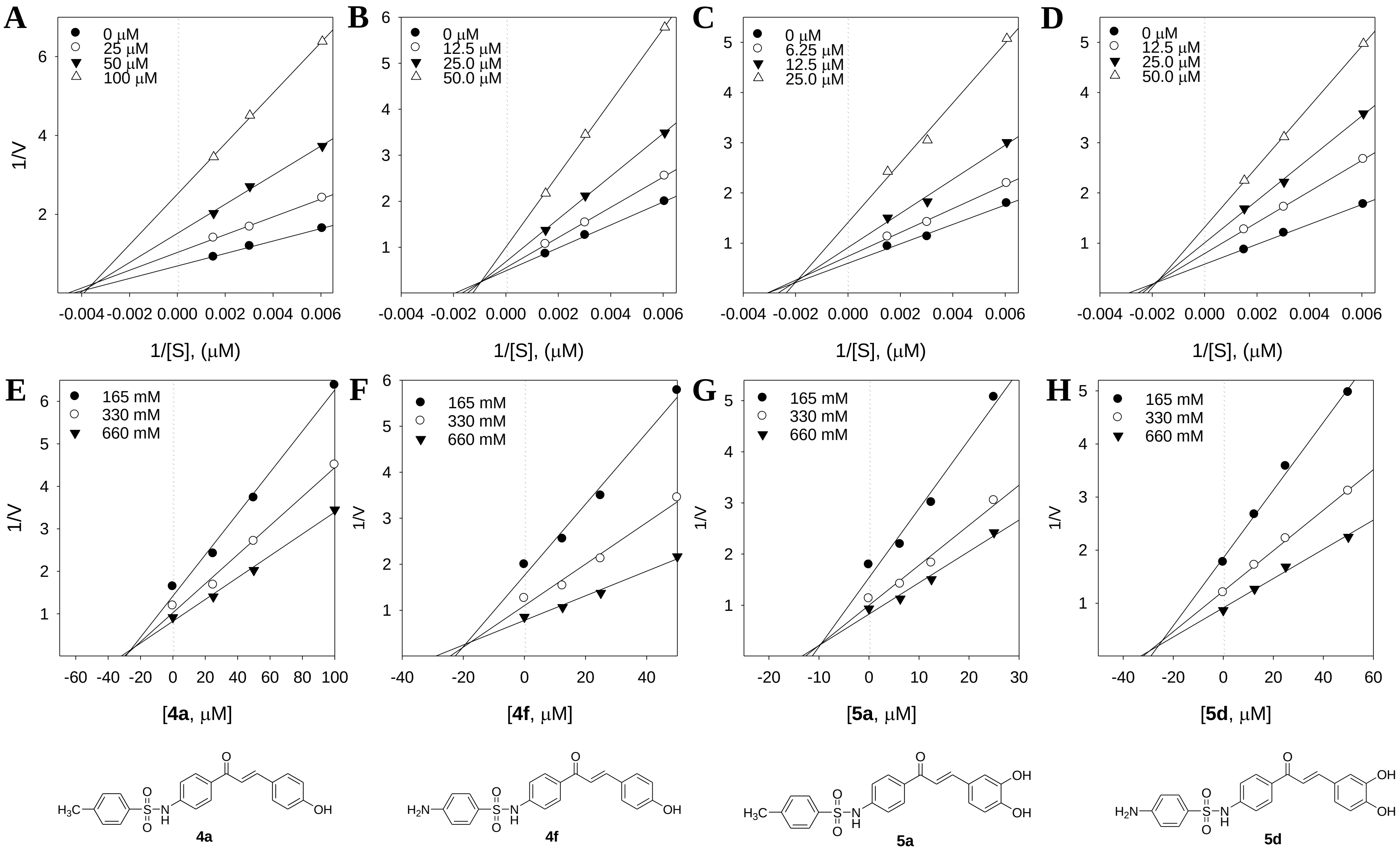

Supplement: Supplementary file 1 [file molecules-18-00140-s001.pdf]
